# The Oxygen Uptake Plateau—A Critical Review of the Frequently Misunderstood Phenomenon

**DOI:** 10.1007/s40279-021-01471-4

**Published:** 2021-04-29

**Authors:** Max Niemeyer, Raphael Knaier, Ralph Beneke

**Affiliations:** 1 Department Medicine, Training and Health, Institute of Sports Science and Motologie, Philipps-University Marburg, Jahnstr. 12, 35037 Marburg, Germany; 2 Division of Sleep Medicine, Harvard Medical School, Boston, MA USA; 3 Medical Chronobiology Program, Division of Sleep and Circadian Disorders, Brigham and Women’s Hospital, Boston, MA USA

## Abstract

A flattening of the oxygen uptake–work rate relationship at severe exercise indicates the achievement of maximum oxygen uptake $\left({\text{VO}}_{2\max } \right)$. Unfortunately, a distinct plateau $\left( {{{\text{VO}}}_{2} {\text{pl}}} \right)$ at ${{\text{VO}}}_{2\max }$is not found in all participants. The aim of this investigation was to critically review the influence of research methods and physiological factors on the ${{\text{VO}}}_{2} {\text{pl}}$ incidence. It is shown that many studies used inappropriate definitions or methodical approaches to check for the occurrence of a ${{\text{VO}}}_{2} {\text{pl}}$. In contrast to the widespread assumptions it is unclear whether there is higher ${{\text{VO}}}_{2} {\text{pl}}$ incidence in (uphill) running compared to cycling exercise or in discontinuous compared to continuous incremental exercise tests. Furthermore, most studies that evaluated the validity of supramaximal verification phases, reported verification bout durations, which are too short to ensure that ${{\text{VO}}}_{2\max }$ have been achieved by all participants. As a result, there is little evidence for a higher ${{\text{VO}}}_{2} {\text{pl}}$ incidence and a corresponding advantage for the diagnoses of ${{\text{VO}}}_{2\max }$ when incremental tests are supplemented by supramaximal verification bouts. Preliminary evidence suggests that the occurrence of a ${{\text{VO}}}_{2} {\text{pl}}$ in continuous incremental tests is determined by physiological factors like anaerobic capacity, ${{\text{VO}}}_{2}$-kinetics and accumulation of metabolites in the submaximal intensity domain. Subsequent studies should take more attention to the use of valid ${{\text{VO}}}_{2} {\text{pl}}$ definitions, which require a cut-off at ~ 50% of the submaximal ${{\text{VO}}}_{2}$ increase and rather large sampling intervals. Furthermore, if verification bouts are used to verify the achievement of ${{\text{VO}}}_{{2{\text{peak}}}}$/${{\text{VO}}}_{2\max }$, it should be ensured that they can be sustained for sufficient durations.

## Key Points

**Table:** 

A near-constant ${\dot{\text{V}}}{{\text{O}}}_{2}$ despite a further increase in work rate (= ${\dot{\text{V}}}{{\text{O}}}_{2} {\text{pl}}$) indicates attainment of ${\dot{\text{V}}}{{\text{O}}}_{2\max }$; however, not all participants demonstrate a ${\dot{\text{V}}}{{\text{O}}}_{2} {\text{pl}}$ at the end of an incremental test.
There is lack of convincing evidence to show that the incidence of the ${\dot{\text{V}}}{{\text{O}}}_{2} {\text{pl}}$ is influenced by exercise mode, exercise protocol, aerobic fitness, anthropometrics, or age.
Preliminary evidence indicates that a fast ${\dot{\text{V}}}{{\text{O}}}_{2}$ -kinetics as well as a high anaerobic capacity and anaerobic threshold related to ${\dot{\text{V}}}{{\text{O}}}_{2\max }$ seem to increase the chance that a plateau at ${\dot{\text{V}}}{{\text{O}}}_{2\max }$ occurs.

## Introduction

**Fig. 1:**
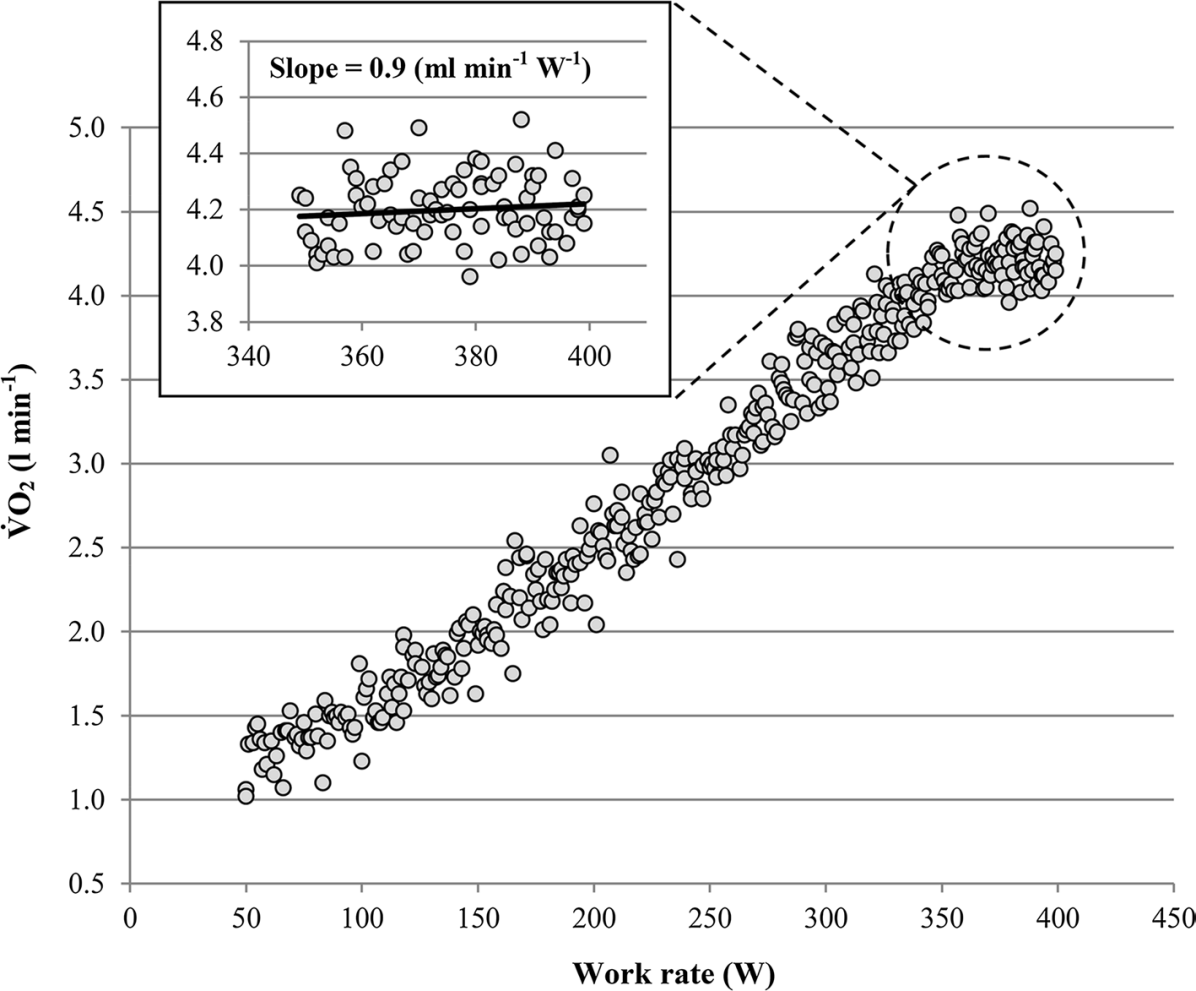
Oxygen uptake (${\dot{\text{V}}}{{\text{O}}}_{2}$) response of a participant with a ${\dot{\text{V}}}{{\text{O}}}_{{2{\text{pl}}}}$ at the end of a continuous incremental ramp test. Note that the slope of the ${\dot{\text{V}}}{{\text{O}}}_{2}$–work rate relationship during the final 50 W is considerably lower than the corresponding slope between 110 and 340 W (= 10.6 mL min^−1^ W^−1^). Slope: slope of the oxygen uptake–work rate relationship of the final 50 W

The first description of the oxygen uptake plateau (${\dot{\text{V}}}{{\text{O}}}_{2} {\text{pl}}$) is attributed to Archibald Vivian Hill [1–3]. The former Nobel Prize laureate and his colleague Hartley Lupton collected their breathing gases with a Douglas bag while running on flat terrain with varying running speeds. In one participant they found that ${\dot{\text{V}}}{{\text{O}}}_{2}$ increased with running speed, but remained constant for speeds beyond 260 m per minute. Hill and Lupton [3] interpreted the flattening of the V̇O_2_–running-speed-relationship as an indication that maximum oxygen uptake (${\dot{\text{V}}}{{\text{O}}}_{2\max }$) has been achieved. In subsequent studies the flattening of the ${\dot{\text{V}}}{{\text{O}}}_{2}$–running-speed-relationship or –work-rate-relationship in the severe-intensity domain, as shown in Fig. 1, has been called “${\dot{\text{V}}}{{\text{O}}}_{2}$-plateau” or “levelling off” [4–7]. Nowadays, ${\dot{\text{V}}}{{\text{O}}}_{2\max }$is considered as one of the most important measurements in exercise physiology and sports medicine [8–10]. However, it has been often ignored that one can be certain that ${\dot{\text{V}}}{{\text{O}}}_{2\max }$ has been reached only if ${\dot{\text{V}}}{{\text{O}}}_{2}$ remains more or less constant despite an increase in work rate. Thus, the diagnosis of ${\dot{\text{V}}}{{\text{O}}}_{2\max }$ requires–per definition–a ${\dot{\text{V}}}{{\text{O}}}_{{2{\text{pl}}}}$, regardless whether the plateau occurs at the end of a continuous incremental test, between the final stages of a discontinuous test or between an incremental test and a subsequent verification test (see Sect. 2.3), as highlighted by Poole and Jones [11].

Unfortunately, only a fraction of participants that undergo exercise testing shows a ${\dot{\text{V}}}{{\text{O}}}_{{2{\text{pl}}}}$. The reported incidences vary between 17 and 94%, even in larger studies with more than 50 participants, especially depending on the used ${\dot{\text{V}}}{{\text{O}}}_{{2{\text{pl}}}}$ definition (see Sect. 2.1) [7, 12–19]. This led to extensive discussions concerning the concept and diagnosis of ${\dot{\text{V}}}{{\text{O}}}_{2\max }$ throughout the last three decades [2, 11, 20–30]. The aim of the present investigation is to critically review the existing body of literature about the ${\dot{\text{V}}}{{\text{O}}}_{{2{\text{pl}}}}$ and aspects of ${\dot{\text{V}}}{{\text{O}}}_{2\max }$ diagnosis with special respect to methodological and physiological determinants of the incidence of the ${\dot{\text{V}}}{{\text{O}}}_{{2{\text{pl}}}}$.

## Methodological Determinants

Although described for the first time almost a century ago, there is still a lot of confusion about the methodological determinants and the correct diagnosis of the V̇O_2_pl phenomenon [23, 24, 27]. This led to the assumption that the ${\dot{\text{V}}}{{\text{O}}}_{{2{\text{pl}}}}$ is a calculation artefact rather than an indication of a physiological event [20, 24, 31–33]. In contrast, other authors suggested that the absence of a ${\dot{\text{V}}}{{\text{O}}}_{{2{\text{pl}}}}$ is mostly caused by inappropriate exercise modes and protocols or data analysing approaches [11, 14, 34–36]. Potential effects of the data analysing approach, exercise mode and protocol on the V̇O_2_pl incidence will be critically revisited below.

### Plateau Definitions

The first quantitative approach for the diagnosis of the ${\dot{\text{V}}}{{\text{O}}}_{{2{\text{pl}}}}$ was provided by Taylor et al. [7]. They performed discontinuous exercise tests on a treadmill with 3-min durations at a speed of 3.13 m s^−1^. Treadmill inclination was increased by 2.5% from test to test at subsequent testing days. When the increase in ${\dot{\text{V}}\text{O}}_{2}$ between subsequent testing days was less than 150 mL min^−1^ (~ 2.1 mL min^−1^ kg^−1^) a ${\dot{\text{V}}\text{O}}_{{2{\text{pl}}}}$was assumed. This cut-off had been determined based on corresponding treadmill tests in the submaximal intensity domain i.e. two or more steps before a ${\dot{\text{V}}\text{O}}_{{2{\text{pl}}}}$ occurred in a subgroup of 13 participants. They found an average increase in ${\dot{\text{V}}\text{O}}_{2}$ of 299.3 ± 86.5 mL min^−1^ (~ 4.2 ± 1.1 mL min^−1^ kg^−1^) with a range of 159–470 mL min^−1^ (~ 2.2–5.9 mL min^−1^ kg^−1^) per 2.5% grade steps. Based on this finding the authors [7] concluded that if the ${\dot{\text{V}}\text{O}}_{2}$ increases for less than 150 mL min^−1^ or 2.1 mL min^−1^ kg^−1^: “…there is small chance of making an error in deciding that the maximal oxygen intake had been reached”.

The cut-offs of Taylor et al. [7] are still widely used for ${\dot{\text{V}}\text{O}}_{{2{\text{pl}}}}$ diagnoses [23, 25], despite this assumption has been challenged in subsequent studies (see Sect. 2.3.2). But more importantly, these cut-offs had been validated for specific testing conditions only. They were deduced from a discontinuous exercise protocol with bouts of constant running speeds long enough to reflect the complete fast component of steady-state ${\dot{\text{V}}\text{O}}_{2}$-kinetics and a specific increase in oxygen demand per subsequent running bout [37, 38]. Instead of such highly specific testing conditions others combined the Taylor et al. [7] cut-offs with arbitrary time intervals, also called sampling intervals, during various one-time continuous incremental test irrespective of the test-specific ${\dot{\text{V}}\text{O}}_{2}$-response [14, 32, 34, 39–41]. Such modifications may lead to mismatches between presumptions implied by Taylor et al. [7] and test-specific increases in ${\dot{\text{V}}\text{O}}_{2}$ per time or workload increment induced using other testing protocols [38, 42]. For example, sampling intervals of 30 s were widely used to check whether a ${\dot{\text{V}}\text{O}}_{{2{\text{pl}}}}$ occurs or not [14, 40, 43–45]. This means that the average ${\dot{\text{V}}\text{O}}_{2}$ of the last and next-to-last 30 s period of a test were compared. At a ramp test with an incremental rate of 30 W min^−1^ the mean difference in work rate between adjacent 30 s sampling intervals is 15 W leading to a mean increase in ${\dot{\text{V}}\text{O}}_{2}$ between adjacent sampling intervals in the submaximal intensity domain of ~ 150 mL min^−1^ [42, 46]. Here application of the 150 mL min^−1^ cut-off by Taylor et al. [7] may lead to ${\dot{\text{V}}\text{O}}_{{2{\text{pl}}}}$ diagnoses despite no flattening at ${\dot{\text{V}}\text{O}}_{2\max }$ occurs. Consequently, application of such fixed or absolute cut-offs on arbitrarily selected testing protocols and time intervals may provoke high risks of false ${\dot{\text{V}}\text{O}}_{{2{\text{pl}}}}$ diagnoses, as described by Beltrami et al. [32] and Marsh [38]. To avoid such mismatches, relative cut-offs which consider the increase in ${\dot{\text{V}}\text{O}}_{2}$in the submaximal intensity domain of a specific testing protocol are strongly recommended [37, 38]. However, even when a relative cut-off had been used several ${\dot{\text{V}}\text{O}}_{{2{\text{pl}}}}$'s in the submaximal intensity domain had been found by Beltrami et al. [32]. Based on this finding they concluded that the ${\dot{\text{V}}\text{O}}_{{2{\text{pl}}}}$ may reflect a calculation artefact rather than an indication of a true physiological event.

Mathematically a plateau is defined as part of a function with a slope equal to zero. However, the breath-by-breath ${\dot{\text{V}}\text{O}}_{2}$ response shows large variability, which is mainly caused by irregularities in the rate and depth of ventilation [25, 47]. Consequently, even under steady-state conditions, the ${\dot{\text{V}}\text{O}}_{2}$ values of subsequent sampling intervals are never exactly similar (i.e. slope ≠ 0) as shown by Myers et al. [47]. Therefore, if the cut-off is set at a slope equal to zero, potential ${\dot{\text{V}}\text{O}}_{{2{\text{pl}}}}$’s which are caused by the limitation of the body to transport and utilise O_2_, may remain undetected (false-negative ${\dot{\text{V}}\text{O}}_{{2{\text{pl}}}}$ diagnosis). To avoid such false-negative ${\dot{\text{V}}\text{O}}_{{2{\text{pl}}}}$ diagnoses the cut-off must be set at a slope or value which is higher than the ventilation-induced variability of ${\dot{\text{V}}\text{O}}_{2}$ [42].

**Fig. 2:**
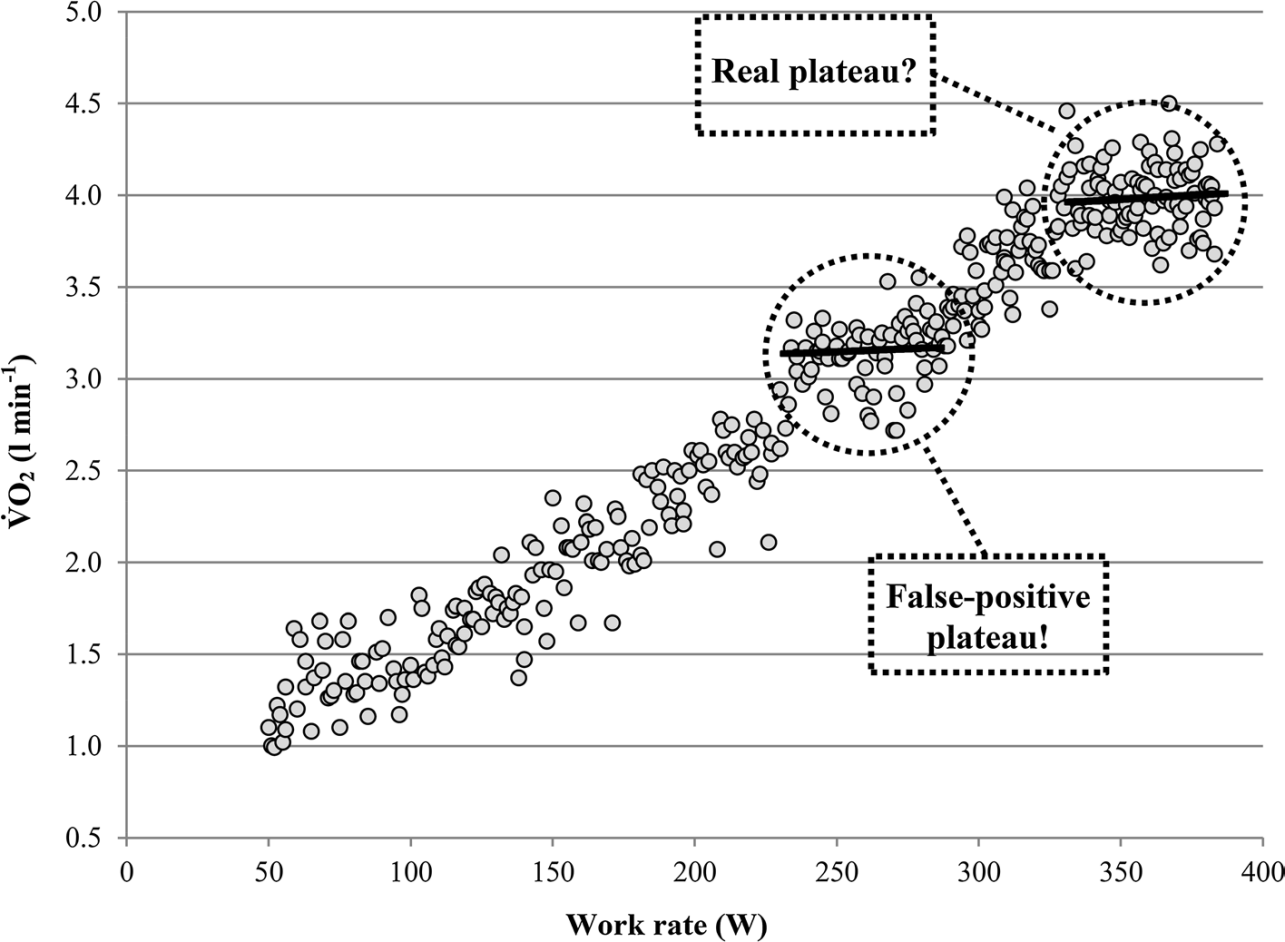
Oxygen uptake (${\dot{\text{V}}\text{O}}_{2}$) ramp test response of a typical participant in which the ventilation-induced variability of ${\dot{\text{V}}\text{O}}_{2}$ may lead to ${\dot{\text{V}}\text{O}}_{{2{\text{pl}}}}$ diagnosis in the submaximal intensity domain. Note that ${\dot{\text{V}}\text{O}}_{{2{\text{pl}}}}$’s in the maximal intensity domain must be more pronounced than plateaus occurring in the submaximal intensity domain to be able to discriminate between false and real plateaus

On the other hand, the variability of ${\dot{\text{V}}\text{O}}_{2}$may result in plateau-like ${\dot{\text{V}}\text{O}}_{2}$-responses in the submaximal intensity domain of incremental exercise tests, as shown in Fig. 2. Such plateau-like ${\dot{\text{V}}\text{O}}_{2}$-responses may result in an erroneously levelling-off-diagnosis if the test shown in Fig. 2 had been terminated prematurely, for example, due to a lack of motivation or pain tolerance, at a work rate of ~ 285 W. Therefore, plateaus in the submaximal intensity domain can be classified as false-positive ${\dot{\text{V}}\text{O}}_{{2{\text{pl}}}}$ diagnoses [32, 42]. To be able to discriminate between plateaus that are simply caused by the variability of ventilation (false-positive ${\dot{\text{V}}\text{O}}_{{2{\text{pl}}}}$’s) and those which are caused by the limitation of the body to transport or utilise O_2_ (real ${\dot{\text{V}}\text{O}}_{{2{\text{pl}}}}$’s), a plateau at ${\dot{\text{V}}\text{O}}_{2\max }$ must be more pronounced than potential plateaus occurring in the submaximal intensity domain [42].

To reduce the risk of false-positive ${\dot{\text{V}}\text{O}}_{{2{\text{pl}}}}$ diagnoses several studies used rather restrictive cut-offs which were set at considerably less than 50% of the mean increase or difference in ${\dot{\text{V}}\text{O}}_{2}$ in the submaximal intensity domain [6, 43–45]. Since the variability of ${\dot{\text{V}}\text{O}}_{2}$ shows a Gaussian distribution [42, 47, 48] and such restrictive cut-offs may be additionally less than the accuracy of common breath-by-breath devices this approach increases the risk of false-negative ${\dot{\text{V}}\text{O}}_{{2{\text{pl}}}}$ diagnoses [2, 42, 47]. Based on data of a recent study [42] this becomes clear. The authors determined the risk of false-positive and false-negative ${\dot{\text{V}}\text{O}}_{{2{\text{pl}}}}$ diagnoses for different work rate defined sampling intervals at a ramp test with an incremental rate of 30 W min^−1^. They started with a sampling interval of 30 W (= 60 s), which means that several slopes were fitted into different 30 W intervals in the submaximal intensity domain to check for the rate/risk of false-positive diagnoses. At this sampling interval the risk of false-positive diagnoses was 12.7%. Since the variability of the slopes was Gaussian distributed it can be calculated that the risk of false-negative diagnoses is also 12.7% if the cut-off is set at half of the increase in ${\dot{\text{V}}\text{O}}_{2}$ in the submaximal intensity domain [42]. If the cut-off is set instead of at one-third of the increase in ${\dot{\text{V}}\text{O}}_{2}$ in the submaximal intensity domain, the risk of false-positive ${\dot{\text{V}}\text{O}}_{{2{\text{pl}}}}$ diagnoses reduces to 6.4%. However, this goes along with a more pronounced increase in the risk of false-negative ${\dot{\text{V}}\text{O}}_{{2{\text{pl}}}}$ diagnoses to 22.4%, such that the combined risk of false-positive and false-negative ${\dot{\text{V}}\text{O}}_{{2{\text{pl}}}}$ diagnoses is slightly higher than if the cut-off set at half of the increase (28.8% vs. 25.4%). This demonstrates that the cut-off should be set at approximately half of the increase in the submaximal intensity domain to enable an equal risk of false-positive and false-negative ${\dot{\text{V}}\text{O}}_{{2{\text{pl}}}}$ diagnoses [42].

However, a combined risk of false ${\dot{\text{V}}\text{O}}_{{2{\text{pl}}}}$ diagnoses of ~ 25% is quite high and do not allow to detect a real ${\dot{\text{V}}\text{O}}_{{2{\text{pl}}}}$ with sufficient certainty. Therefore, the sampling interval was subsequently increased to 40 (= 80 s) and 50 W (= 100 s), which led to a reduction of the combined risk of false-positive and false-negative ${\dot{\text{V}}\text{O}}_{{2{\text{pl}}}}$ to ~ 9.4 (40 W) and ~ 3.2% (50 W) [42]. Application of the latter ${\dot{\text{V}}\text{O}}_{{2{\text{pl}}}}$ definition to the final 50 W increment of the ramp tests resulted in the detection of a considerably higher ${\dot{\text{V}}\text{O}}_{{2{\text{pl}}}}$ incidence (35.7%) than the expected rate of false-positive ${\dot{\text{V}}\text{O}}_{{2{\text{pl}}}}$ due to the variability of ${\dot{\text{V}}\text{O}}_{2}$ (1.6%) [42]. This indicates that a ${\dot{\text{V}}\text{O}}_{{2{\text{pl}}}}$ in terms of a real change in the VO_2_/P-ratio at ${\dot{\text{V}}\text{O}}_{2\max }$ exists which is not simply caused by the variability of ventilation or a calculation artefact as suggested by some researchers [20, 24, 31–33, 47]. However, the observed ${\dot{\text{V}}\text{O}}_{{2{\text{pl}}}}$ incidence at maximal intensity of ~ 35% in the Niemeyer et al. [42] study was much lower than the corresponding incidences of 90–100% reported occasionally [14, 34]. Since the latter studies used very short sampling intervals combined with inappropriate absolute cut-offs these extremely high ${\dot{\text{V}}\text{O}}_{{2{\text{pl}}}}$ incidences likely reflect a high fraction of false-positive ${\dot{\text{V}}\text{O}}_{{2{\text{pl}}}}$ diagnoses [32, 38, 42]. Therefore, assumptions [14, 34] that the absence of a ${\dot{\text{V}}\text{O}}_{{2{\text{pl}}}}$ is caused by an inappropriate plateau definition and/or insufficient data analyses methodology appear unfounded. V̇O_2_pl incidences of about 20–60% seem realistic if more appropriate ${\dot{\text{V}}\text{O}}_{{2{\text{pl}}}}$ definitions are used [13, 18, 19, 42, 49–52].

With respect to the written above, an appropriate ${\dot{\text{V}}\text{O}}_{{2{\text{pl}}}}$ definition requires (1) that the cut-off is set at approximately 50% of the increase in ${\dot{\text{V}}\text{O}}_{2}$ in the submaximal intensity domain of a specific testing protocol; (2) that rather large sampling intervals are used to check for the occurrence of a ${\dot{\text{V}}\text{O}}_{{2{\text{pl}}}}$. For ramp exercise tests with incremental rates of 30 ± 10 W min^−1^ sampling intervals of approximately 40–50 W seems to be ideal [42]. This means that the increase in ${\dot{\text{V}}\text{O}}_{2}$ during the final 40–50 W of an incremental exercise test must be less than half of the increase in the submaximal intensity domain to be certain that a real ${\dot{\text{V}}\text{O}}_{{2{\text{pl}}}}$ plateau occurs [42]. Importantly, this approach cannot be transferred to incremental tests with much lower incremental rates which are usually applied to sedentary or clinical populations, as highlighted by Niemeyer et al. [42]. The same applies to other exercise modes, such as rowing or running.

As a result, it is impossible to evaluate the validity of the plateau definitions of all published studies which are relevant for the present review with sufficient certainty. This applies also to studies that did not report sampling intervals or incremental rates. Therefore, we opted against to exclude studies that used ${\dot{\text{V}}\text{O}}_{{2{\text{pl}}}}$ definitions with unclear validity. Instead, potential effects of the used ${\dot{\text{V}}\text{O}}_{{2{\text{pl}}}}$ definitions on the corresponding findings in the subsequent sections are discussed. Studies that used rather large sampling intervals (i.e. the ${\dot{\text{V}}\text{O}}_{{2{\text{pl}}}}$ was determined from more than the final 30 W or 60 s) and adequate cut-offs were classified as “probably valid”. An adequate cut-off was assumed when the cut-off was derived from the increase in ${\dot{\text{V}}\text{O}}_{2}$ in the submaximal intensity domain or set at approximately half of the expected increase in ${\dot{\text{V}}\text{O}}_{2}$in the submaximal intensity domain.

### Exercise Mode

It is well-known that ${\dot{\text{V}}\text{O}}_{2\max } /{\dot{\text{V}}\text{O}}_{{2{\text{peak}}}}$ varies between exercise modes. Untrained or non-specifically trained participants achieve a 5–15% higher ${\dot{\text{V}}\text{O}}_{2\max } /{\dot{\text{V}}\text{O}}_{{2{\text{peak}}}}$ in uphill treadmill running than in cycling or other exercise modes [5, 53–56]. Only specifically trained athletes may reach higher or comparable ${\dot{\text{V}}\text{O}}_{2\max } /{\dot{\text{V}}\text{O}}_{{2{\text{peak}}}}$ in their accustomed discipline than in (uphill) running [56, 57]. Based on the assumption that a ${\dot{\text{V}}\text{O}}_{{2{\text{pl}}}}$ occurs only at the highest rate of oxygen uptake that participants can attain when the mode fits perfectly to their abilities (= ${\dot{\text{V}}\text{O}}_{2\max }$), it is widely believed that there is a higher ${\dot{\text{V}}\text{O}}_{{2{\text{pl}}}}$ incidence in (uphill) running or in the specifically trained discipline compared to other exercise modes [27, 35, 36, 58]. However, only two studies analysed ${\dot{\text{V}}\text{O}}_{{2{\text{pl}}}}$ incidences at different exercise modes based on within-subject designs [39, 44]. Both studies described significantly higher ${\dot{\text{V}}\text{O}}_{{2{\text{pl}}}}$ incidences in uphill treadmill running (~ 50%) than in cycling (8% and 20%, respectively). However, both studies used absolute cut-offs for the diagnoses of a ${\dot{\text{V}}\text{O}}_{{2{\text{pl}}}}$ and did not account for different increases in ${\dot{\text{V}}\text{O}}_{2}$ between exercise modes and specific testing protocols, respectively. For example, Gordon et al. [44] used an incremental rate of 0.5 W s^−1^ for cycling leading to an increase in ${\dot{\text{V}}\text{O}}_{2}$ in the submaximal intensity domain between consecutive 30 s sampling intervals of about ~ 150 mL min^−1^ [42]. In contrast, the incremental rate of 0.5% per 30 s-interval at a running speed of 10 km h^−1^ for the treadmill test led to an increase in ${\dot{\text{V}}\text{O}}_{2}$ of about ~ 60 mL min^−1^ between consecutive 30 s sampling intervals. Since the cut-off was set at 50 mL min^−1^ for both exercise modes, there was a much higher risk of false-positive ${\dot{\text{V}}\text{O}}_{{2{\text{pl}}}}$ diagnoses at the treadmill compared to the cycling tests. A similar problem applies to the study by Rivera-Brown et al. [39]. Consequently, based on the present evidence it is unclear whether there is really a higher ${\dot{\text{V}}\text{O}}_{{2{\text{pl}}}}$ incidence in (uphill) running compared to other exercise modes.

Irrespective of this, it has been shown that participants may demonstrate a clear ${\dot{\text{V}}\text{O}}_{{2{\text{pl}}}}$ at the end of incremental cycling tests despite a higher ${\dot{\text{V}}\text{O}}_{2\max }$ during treadmill or combined leg and arm cycling tests [59–61]. Therefore, a ${\dot{\text{V}}\text{O}}_{{2{\text{pl}}}}$ indicates only the achievement of a mode- or task-specific ${\dot{\text{V}}\text{O}}_{2\max }$ and not the achievement of the highest rate of oxygen uptake that a participant can attain when most of the skeletal muscles are activated and/or the exercise mode corresponds to the training specificity [61].

### Exercise Protocol

#### Exercise Duration and Incremental Rate

The effect of different incremental rates and the resulting time to exhaustions on ${\dot{\text{V}}\text{O}}_{2\max } /{\dot{\text{V}}\text{O}}_{{2{\text{peak}}}}$ has been observed in several studies. Some of them suggest that ${\dot{\text{V}}\text{O}}_{2\max } /{\dot{\text{V}}\text{O}}_{{2{\text{peak}}}}$ can be measured in a wide range of incremental rates leading to exhaustion between approximately 5 and 25 min [62, 63]. However, other studies showed that an incremental rate should be used, which leads to exhaustion between 8 and 12 or 16 min [64–67]. To the best of our knowledge, there is only one study with a within-subject design which checked the effect of incremental rate on the ${\dot{\text{V}}\text{O}}_{{2{\text{pl}}}}$ incidences [64]. In this study incremental rates between 15 and 90 W min^−1^ were applied to 16 trained participants (eight men and eight women) to accomplish ramp test durations of approximately 5, 8, 12 and 16 min. The authors [64] did not find any systematic effects of the test durations or related incremental rates to the incidence of the ${\dot{\text{V}}\text{O}}_{{2{\text{pl}}}}$. The highest incidences of the ${\dot{\text{V}}\text{O}}_{{2{\text{pl}}}}$ were found at ramp tests of 8 and 16 min duration. Unfortunately, the authors [64] defined a ${\dot{\text{V}}\text{O}}_{{2{\text{pl}}}}$ as an increase in ${\dot{\text{V}}\text{O}}_{2}$ of < 50 mL min^−1^ within the final 30 s disregarding the fact that the expected increases in ${\dot{\text{V}}\text{O}}_{2}$ in the submaximal intensity domain ranged from 75 mL min^−1^ to 450 mL min^−1^ between the different protocols. Thus, a much higher risk of false-positive ${\dot{\text{V}}\text{O}}_{{2{\text{pl}}}}$ diagnoses in those protocols with lower incremental rates has to be expected (see Sect. 2.1).

The combined findings from studies using either high or low incremental rates indicate that ${\dot{\text{V}}\text{O}}_{{2{\text{pl}}}}$ occur in a wide range of incremental rates and corresponding test durations. Thus, ${\dot{\text{V}}\text{O}}_{{2{\text{pl}}}}$ were reported in incremental tests leading to exhaustion within ~ 8 but also ~ 24 min [49, 68–70]. This indicates that ${\dot{\text{V}}\text{O}}_{{2{\text{pl}}}}$ occur within the same range of incremental rates and resulting test durations which enables the achievement of ${\dot{\text{V}}\text{O}}_{2\max }$. However, further studies using appropriate plateau definitions are needed to verify whether there is an optimal incremental rate or test duration for the ${\dot{\text{V}}\text{O}}_{{2{\text{pl}}}}$ occurrence.

#### Continuous vs. Discontinuous Exercise

**Fig. 3:**
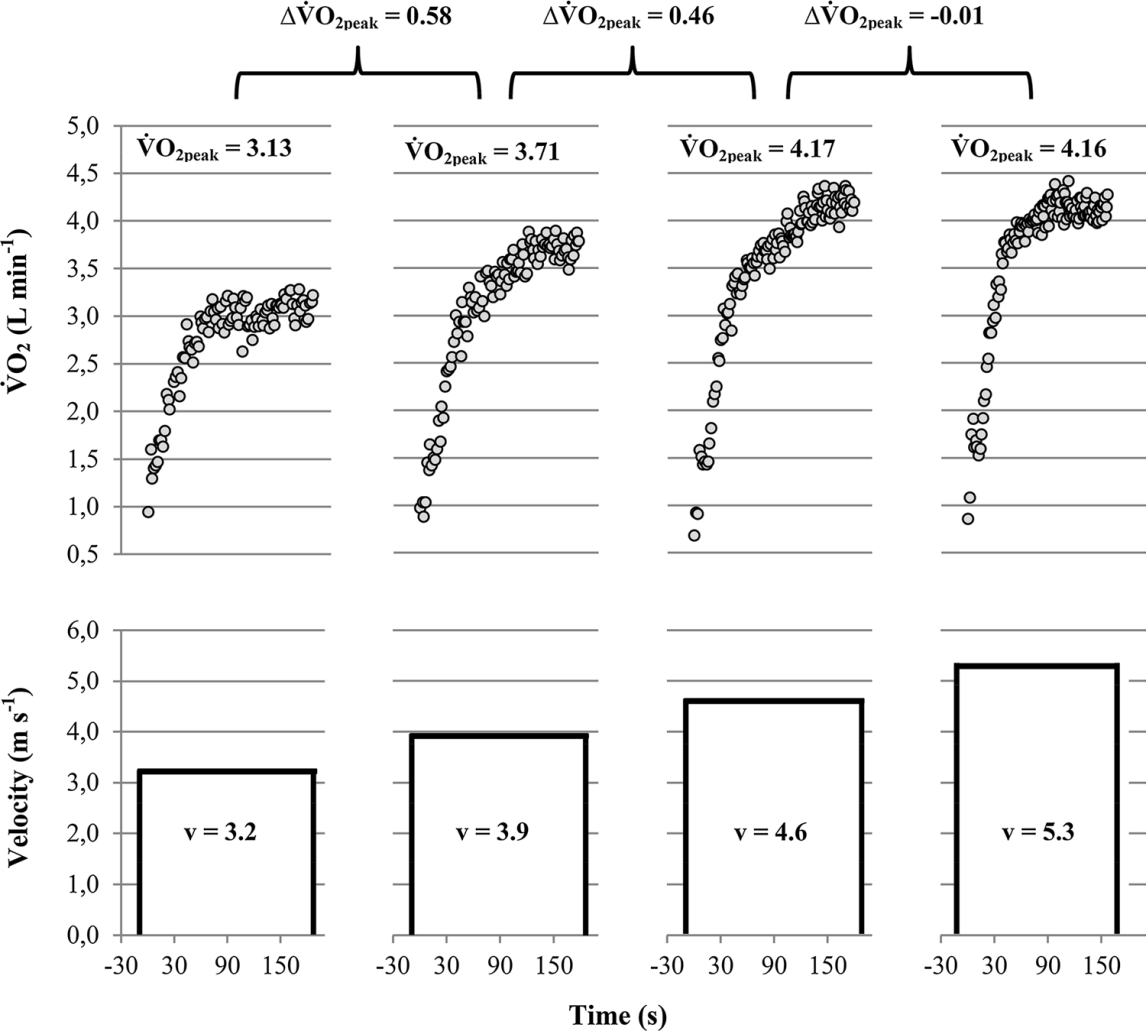
Oxygen uptake (${\dot{\text{V}}\text{O}}_{2}$) (above) and velocity (below) profile of a discontinuous running test performed on separated days at running speeds of 3.2, 3.9, 4.6, and 5.3 m s^−1^. Note that peak oxygen uptake (${\dot{\text{V}}\text{O}}_{{2{\text{peak}}}}$) between the 4.6 and 5.3 m s-^1^ running bouts did not increase any further despite an increase in running speed of 0.7 m s^−1^, which indicates the achievement of a ${\dot{\text{V}}\text{O}}_{{2{\text{pl}}}}$. ∆${\dot{\text{V}}\text{O}}_{{2{\text{peak}}}}$: difference between the peak oxygen uptake values achieved at the separate running bouts, ${\dot{\text{V}}\text{O}}_{{2{\text{pl}}}}$: oxygen uptake plateau. ${\dot{\text{V}}\text{O}}_{{2{\text{pl}}}}$ was calculated as the mean of the highest 30 s-interval of each running bout

Early studies used discontinuous exercise tests to measure ${\dot{\text{V}}\text{O}}_{2\max }$ [3, 6, 7]. As shown in Fig. 3 discontinuous tests are characterised by several discrete constant load exercises, which are separated by long resting periods (i.e. hours or days). For prior applications of discontinuous protocols used to measure${\dot{\text{V}}\text{O}}_{2\max }$, the intensity of the constant load bouts was increased until the corresponding increase in ${\dot{\text{V}}\text{O}}_{2}$ fell short of a predefined cut-off or until the work rate could not be sustained for a predefined duration [5–7, 52]. Due to their time-consuming nature and improvements in measurement equipment, discontinuous protocols have been replaced by continuous incremental exercise protocols.

As shown in several studies ${\dot{\text{V}}\text{O}}_{2\max } /{\dot{\text{V}}\text{O}}_{{2{\text{peak}}}}$ does not differ between discontinuous and continuous exercise tests [52, 53, 55, 71]. However, compared to the discontinuous test protocol used by Taylor et al. [7] ${\dot{\text{V}}\text{O}}_{{2{\text{pl}}}}$incidences are considerably lower in most studies using continuous tests [12, 13, 15, 18, 19, 51]. This led to the assumption that ${\dot{\text{V}}\text{O}}_{{2{\text{pl}}}}$incidences are lower in continuous compared to discontinuous exercise tests [2, 11, 13]. To the best of our knowledge, there is only one study that compared ${\dot{\text{V}}\text{O}}_{{2{\text{pl}}}}$ incidences of continuous and discontinuous exercise tests using a within-subject design [52]. In this study the original discontinuous exercise protocol of Taylor et al. [7], and a continuous incremental treadmill test with an increase of treadmill grade of 2.5% every minute, were used to measure ${\dot{\text{V}}\text{O}}_{2\max }$. In contrast to the study of Taylor et al. [7], Duncan et al. [52] increased the treadmill grade not only until the increase in ${\dot{\text{V}}\text{O}}_{2}$ between subsequent treadmill grades was < 2.1 mL min^−1^ kg^−1^, but until the participants could not sustain the discontinuous exercises for 3 min. Analysing the final two 2.5% grade steps with the cut-off defined by Taylor et al. [7], Duncan et al. [52] found a ${\dot{\text{V}}\text{O}}_{{2{\text{pl}}}}$ incidence of 60% and 50% at the discontinuous and continuous tests, respectively. However, if they accepted a ${\dot{\text{V}}\text{O}}_{{2{\text{pl}}}}$ as soon as the increase in ${\dot{\text{V}}\text{O}}_{2}$ between consecutive 2.5% discontinuous steps was less than the cut-off as implemented by Taylor et al. [7] the ${\dot{\text{V}}\text{O}}_{{2{\text{pl}}}}$ incidence in the discontinuous test was 80%. This finding is supported by an early study [5] which showed that the original approach of Taylor et al. [7] leads to several false-positive ${\dot{\text{V}}\text{O}}_{{2{\text{pl}}}}$ diagnoses. Also, other studies [58, 72, 73] using discontinuous exercise tests identified far lower incidences of ${\dot{\text{V}}\text{O}}_{{2{\text{pl}}}}$ than Taylor et al. [7], however, those numbers were in the range of continuous test studies [18, 19, 42, 50, 51]. In conclusion, there is no convincing evidence for a higher ${\dot{\text{V}}\text{O}}_{{2{\text{pl}}}}$ incidence in discontinuous compared to continuous exercise tests. The extremely high ${\dot{\text{V}}\text{O}}_{{2{\text{pl}}}}$ incidences described by Taylor et al. [7] are likely caused by the specific approach of that study, which was likely to result in a high rate of false-positive ${\dot{\text{V}}\text{O}}_{{2{\text{pl}}}}$ diagnoses [5, 52]. Since the only study [52] that compared the ${\dot{\text{V}}\text{O}}_{{2{\text{pl}}}}$ incidence between discontinuous and continuous tests with a within-subject design had a rather small sample size (*n* = 10) further studies are needed to verify whether the ${\dot{\text{V}}\text{O}}_{{2{\text{pl}}}}$ incidence differs between discontinuous and continuous exercise protocols.

#### Combination of Continuous and Discontinuous Exercise: The Verification Phase

**Fig. 4:**
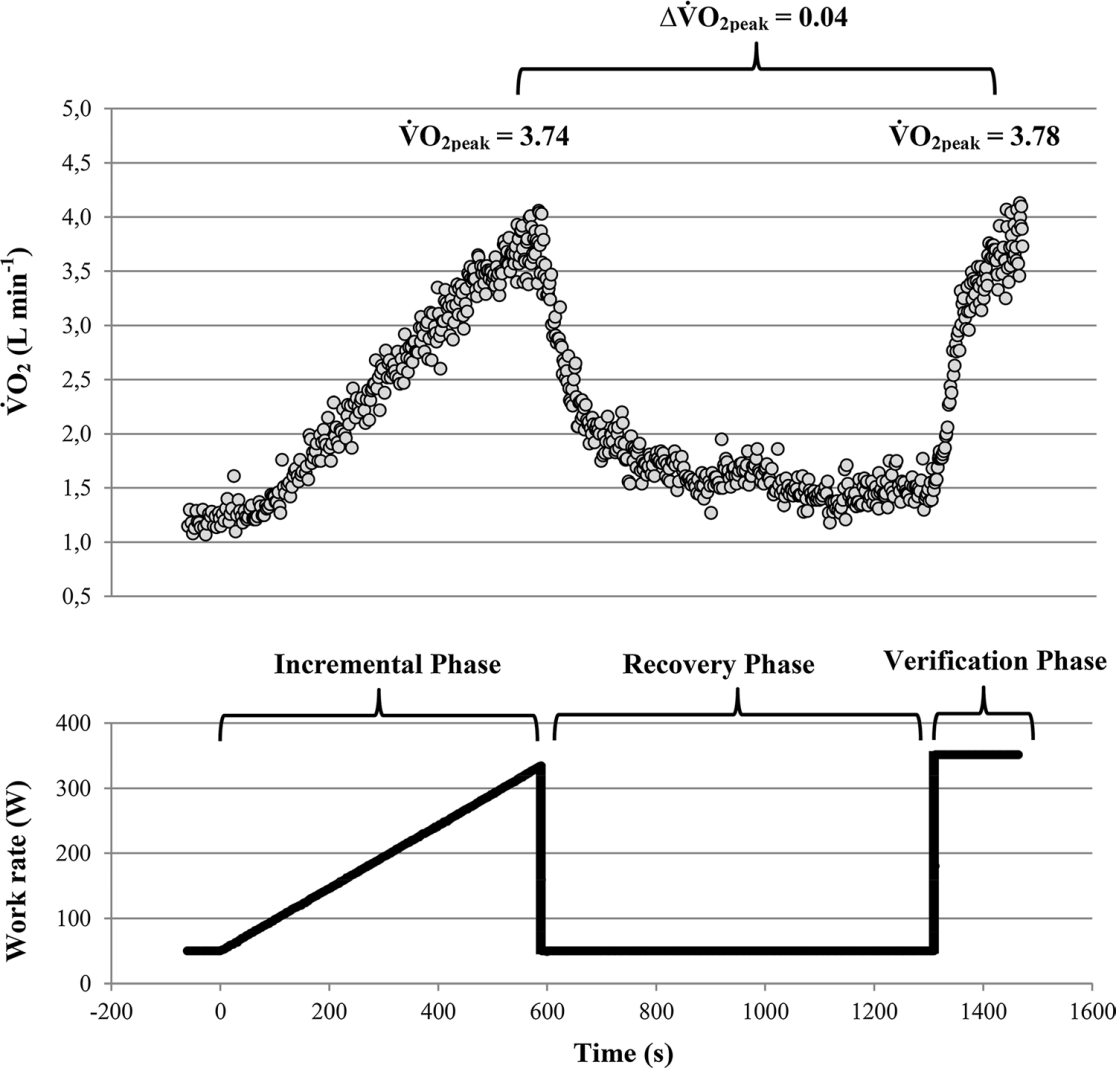
Oxygen uptake (${\dot{\text{V}}\text{O}}_{2}$) (above) and work rate (below) profile of an incremental ramp test, which was followed by a recovery and a verification phase. Note that peak oxygen uptake (${\dot{\text{V}}\text{O}}_{{2{\text{peak}}}}$) of the ramp and verification phases were nearly similar despite the verification bout was performed at a 5% higher work rate than maximum work rate of the ramp bout, which indicates the achievement of a ${\dot{\text{V}}\text{O}}_{{2{\text{pl}}}}$. ∆${\dot{\text{V}}\text{O}}_{{2{\text{pl}}}}$: difference between the peak oxygen uptake values achieved at the incremental and verification phase. ${\dot{\text{V}}\text{O}}_{{2{\text{pl}}}}$ was calculated as the mean of the highest 30 s-interval of the incremental and verification bout, respectively

Based on low ${\dot{\text{V}}\text{O}}_{{2{\text{pl}}}}$ incidences and questionable validity of secondary exhaustion criteria the use of ${\dot{\text{V}}\text{O}}_{2\max }$ verification tests/phases are strongly recommended by some researchers [11, 74]. As shown in Fig. 4 this approach includes a constant-load exercise test, which is performed after a common continuous incremental load test and a recovery phase. If ${\dot{\text{V}}\text{O}}_{{2{\text{peak}}}}$ of the incremental and verification phases do not differ despite the verification bout was performed on a higher work rate than maximum work rate of the incremental test i.e. supramaximal work rate (related to maximum work rate at termination of the incremental test), a ${\dot{\text{V}}\text{O}}_{{2{\text{pl}}}}$ occurs and ${\dot{\text{V}}\text{O}}_{2\max }$ of the incremental test is considered as verified [11]. If ${\dot{\text{V}}\text{O}}_{{2{\text{peak}}}}$ of the verification phase is higher than ${\dot{\text{V}}\text{O}}_{{2{\text{peak}}}}$ of the incremental test no indication of a ${\dot{\text{V}}\text{O}}_{{2{\text{pl}}}}$ has been verified and ${\dot{\text{V}}\text{O}}_{2\max }$ of the incremental test is considered as falsified [11]. Several studies reported considerable lower plateau occurrences during the final part of a single incremental test as compared to the difference between the ${\dot{\text{V}}\text{O}}_{{2{\text{peak}}}}$ values of an incremental test and the ${\dot{\text{V}}\text{O}}_{{2{\text{peak}}}}$ values of a subsequent supramaximal verification bout [69, 75–79].

However, besides a supramaximal work rate of the verification bout [11], there is another important prerequisite for valid ${\dot{\text{V}}\text{O}}_{{2{\text{pl}}}}$ diagnoses based on the combination of incremental and verification phases. Because of a rather slow ${\dot{\text{V}}\text{O}}_{2}$-kinetics, the duration of the verification bout must be long enough to reach ${\dot{\text{V}}\text{O}}_{2\max }$ [11, 28]. According to Hill et al. [80], the minimum duration of an exhaustive exercise trial to rise ${\dot{\text{V}}\text{O}}_{2}$to its maximum value is 2.3 ± 0.3 min. A more detailed study of Caputo and Denadai [81] showed that in untrained and non-specifically trained participants durations of at least 3.5 ± 0.5 and 2.8 ± 0.5 min are required, respectively. In contrast, in specifically endurance-trained athletes durations of 2.0 ± 0.5 min were sufficiently long to rise ${\dot{\text{V}}\text{O}}_{2}$ to its maximum value [81]. These minimum durations are supported by ${\dot{\text{V}}\text{O}}_{2}$ kinetic studies, which showed that endurance-trained participants have considerable shorter time constants of ${\dot{\text{V}}\text{O}}_{2}$ kinetics compared to non-specific or untrained individuals [82–84]. Thus, to construct a real ${\dot{\text{V}}\text{O}}_{{2{\text{pl}}}}$ between incremental and verification phases, the work rate of the verification bout must be at supramaximal load and needs to be sustained for a minimum of ~ 2 (trained), ~ 3 (non-specifically trained) or ~ 3.5 (untrained) minutes. Since heavy/severe prior exercise leads to speeding of ${\dot{\text{V}}\text{O}}_{2}$ kinetics [85, 86] it seems to be likely that ${\dot{\text{V}}\text{O}}_{2\max }$ will be achieved on average slightly earlier in verification bouts, which are performed a few minutes (up to ~ 30 min) after incremental tests. However, these numbers represent the mean values of the corresponding cohorts, which mean that some participants need even longer to achieve ${\dot{\text{V}}\text{O}}_{2\max }$. These durations should be therefore viewed as an absolute minimum, even if the verification bout is performed in a primed state.

Table 1 summarises published incremental rates, ${\dot{\text{V}}\text{O}}_{2\max }$, and time-to-exhaustions (TTE) of studies which evaluated the use of supramaximal verification tests and reported exact TTE values [37, 68–70, 76, 77, 79, 87–94]. Based on this table it becomes clear that only three out of the 22 studies or sub-studies reported mean TTE values of verification bouts of sufficient duration to achieve ${\dot{\text{V}}\text{O}}_{2\max }$ with convincing probability [70, 77, 87]. The study reporting the longest TTE performed a supramaximal verification bout at 105% of the work rate at the termination of a common incremental test and a recovery phase of at least 24 h in a group of untrained participants [87]. The TTE of 4.2 ± 2.0 was more than 100% higher than the mean TTE of all other cycling studies performing a verification bout at 105% (see Table 1). However, the large standard deviation of the TTE of the verification bout indicates that a considerable fraction of participants did not reach the required minimum test duration for untrained participants of > 3:30 min.

**Table 1:** Summary of the most important findings of studies or sub-studies that evaluated the validity of supramaximal ${\dot{\text{V}}\text{O}}_{2\max }$ verification testing in healthy participants sorted by time to exhaustion (TTE) of the verification bout

Authors (year)	Subjects (*n*)	Exercise mode	Incremental test				Verification test		
			Incremental rate	TTE (min)	${\dot{\text{V}}\text{O}}_{2\max }$ (L min^−1^/ mL min^−1^ kg^−1^)	Recovery duration	Intensity (%P_max_)	TTE (min)	${\dot{\text{V}}\text{O}}_{2\max }$ (L min^−1^/ mL min^−1^ kg^−1^)
**Astorino et al.** [87]	**Untrained** **(*n*** **= 15)**	**Cycling**	**14–21** **(W min**^**−1**^**)**	**9.2 ± 2.4**	**2.37 ± 0.69**	** ≥ 24 (h)**	**105**	**4.2 ± 2.0**	**2.29 ± 0.75**
**Sanchez-Otero et al.** [70]	**Specific trained** **(*n*** **= 12)**	**Running**	**0.14** **(m s**^**−1**^ **min**^**−1**^**)**	**23.9 ± 2.1**	**59.4 ± 5.1**	**15(min)**	** ~ 105.3**	**3.0 ± 0.6**	56.2 ± 4.7*
**Midgley et al.** [77]	**Specific trained** **(*n*** **= 16)**	**Running**	**0.28/0.14** **(m s**^**−1**^ **min**^**−1**^**)**	**11.6 ± 1.9**	**4.04 ± 0.46**	**10 (min)**	**~ 102–103**	**2.8 ± 0.6**	**3.99 ± 0.45**
Scharhag-Rosenberger et al. [79]	Non-specific trained (*n* = 40)	Running	0.19 (m s^−1^ min^−1^)	15.0 ± 2.0	3.82 ± 0.99	≥ 24 (h)	110	2.7 ± 0.6	3.75 ± 1.0
Nolan et al. [90]	Non-specific trained (*n* = 12)	Running	1 (% min^−1^)	11.0 ± 1.8	56.9 ± 9.6	20 (min)	105	2.6 ± 0.6	57.2 ± 9.0
Nolan et al. [90]	Non-specific trained (*n* = 12)	Running	1 (% min^−1^)	11.4 ± 2.4	56.2 ± 9.0	60 (min)	105	2.4 ± 0.7	56.2 ± 9.1
Scharhag-Rosenberger et al. [79]	Non-specific trained (*n* = 40)	Running	0.19 (m s^−1^ min^−1^)	15.0 ± 2.0	3.82 ± 0.99	10 (min)	110	2.1 ± 0.4	3.72 ± 0.99
Astorino et al. [88]	Non-specific trained (*n* = 30)	Cycling	23–29 (W min^−1^)	7.7 ± 1.48	2.90 ± 0.60	10 (min)	~ 106–109	2.1 ± 0.9	2.90 ± 0.60
Astorino et al. [87]	Untrained (*n* = 9)	Cycling	15–30 (W min^−1^)	10.9 ± 1.26	2.67 ± 0.60	1–1.5 (h)	115	2.0 ± 0.4	2.75 ± 0.76
Keiller et al. [76]	Non-specific trained (*n* = 11)	Running	1 (% min^−1^)	8.8 ± 1.4	52.7 ± 5.64	6 (min)	~ 110–112	1.9 ± 0.4	49.7 ± 4.30*
Astorino et al. [89]	Non-specific trained (*n* = 79)	Cycling	6.7 (W min^−1^)	25.6 ± 3.6	2.82 ± 0.62	10 (min)	110	1.9 ± 0.4	2.78 ± 0.59
McGawley et al. [68]	Specific trained (*n* = 10)	Running	1 (% min^−1^)	8.0 ± 0.9	59.2 ± 6.8	9 (min)	105	1.8 ± 0.3	58.1 ± 6.7*
Nolan et al. [90]	Non-specific trained (*n* = 12)	Running	1 (% min^−1^)	11.2 ± 1.2	57.5 ± 9.2	20 (min)	115	1.8 ± 0.5	56.9 ± 9.6
Midgley et al. [37]	Specific trained (*n* = 10)	Cycling	30 (W min^−1^)	10.7 ± 1.3	4.05 ± 0.47	10 (min)	~ 108	~ 1.7	3.96 ± 0.38
Murias et al. [91]	Untrained (*n* = 45)	Cycling	15–25 (W min^−1^)	~ 10.5	40.6 ± 11.4	5 (min)	105	1.7 ± 0.4	40.9 ± 10.9
Astorino et al. [89]	Non-specific trained (*n* = 30)	Cycling	20–40 (W min^−1^)	10.1 ± 1.3	3.35 ± 1.01	8 (min)	105	1.7 ± 0.4	3.32 ± 1.00
Nolan et al. [90]	Non-specific trained (*n* = 12)	Running	1 (% min^−1^)	11.2 ± 1.5	57.1 ± 8.4	60 (min)	115	1.7 ± 0.7	56.0 ± 9.3
Sansum et al. [69]	Untrained children (*n* = 128)	Cycling	10–30 (W min^−1^)	9.5 ± 2.1 (m) 8.1 ± 2.0 (f)	2.48 ± 0.73 (m) 1.96 ± 0.31 (f)	~ 30 (min)	105/110	1.6 ± 0.42 (m) 1.7 ± 0.33 (f)	2.36 ± 0.72 (m)* 1.89 ± 0.34 (f)*
Rossiter et al. [92]	Non-specific trained (*n* = 7)	Cycling	20 (W min^−1^)	~ 16.1	4.33 ± 0.52	5 (min)	105	1.5 ± 0.3	4.30 ± 0.51
Midgley et al. [37]	Specific trained (*n* = 10)	Running	0.28 (m s^−1^ min^−1^)	11.4 ± 0.8	3.86 ± 0.39	10 (min)	~ 105–107	1.5	3.92 ± 0.46
Barker et al. [93]	Untrained children (*n* = 13)	Cycling	10 (W min^−1^)	10.9 ± 1.5	1.69 ± 0.28	15 (min)	105	1.5 ± 0.4	1.62 ± 0.31
Sedgeman et al. [94]	Non-specific trained (*n* = 13)	Cycling	Not mentioned	11.0 ± 1.7	50.1 ± 6.8	3 (min)	105	1.3 ± 0.4	49.1 ± 6.7

Note that the bold indicates the studies with a sufficient duration of the verification bout (untrained > 3.5 min, non-specifically trained > 3.0 min, and specific endurance-trained > 2.0 min). *TTE *time to exhaustion, ${\dot{\text{V}}\text{O}}_{2\max }$ maximum oxygen uptake, *m* male subgroup, *f *female subgroup, *Significant difference between verification and incremental test ${\dot{\text{V}}\text{O}}_{2\max }$ Studies that reported separate findings of subgroups, e.g. because they performed in one subgroup treadmill tests and in the other one cycling tests, were presented for each subgroup separately

The two other studies that reported mean TTE values of sufficient duration were performed on treadmills [70, 77]. One of them [70] used an incremental test with a rather low incremental rate which led to a more than twice as large incremental test durations (23.9 ± 2.1 min) compared to most other studies (see Table 1). In incremental tests the maximally achievable work rate or running speed is positively related to the incremental rate [62, 67]. Therefore, the work rate of a verification bout which is performed at a given percentage of maximum work rate or running speed depends on the incremental rate of the preceding incremental test. At a first glance, this seems to support the use of low incremental rates to guarantee that subsequent verification bouts can be sustained for sufficient durations. However, this benefit comes with the risk to reduce the chance that ${\dot{\text{V}}\text{O}}_{2\max }$ will be achieved in the incremental phase as the optimal duration for ${\dot{\text{V}}\text{O}}_{2\max }$ testing may be exceeded [64–67]. Interestingly, the study of Sanchez-Otero et al. [70], described a significantly lower ${\dot{\text{V}}\text{O}}_{{2{\text{peak}}}}$ in the verification bout compared to the incremental test despite the verification bout lasted long enough. This is potentially caused by the phenomenon that well-trained athletes do not reach ${\dot{\text{V}}\text{O}}_{2\max }$ by fast component ${\dot{\text{V}}\text{O}}_{2}$ response during square-wave exercise [95–97], which further limits the validity of supramaximal verification testing to increase the ${\dot{\text{V}}\text{O}}_{{2{\text{pl}}}}$ incidence.

Nevertheless, most other studies reported similar mean ${\dot{\text{V}}\text{O}}_{{2{\text{peak}}}}$-values in the incremental and verification phases (see Table 1). However, this does not indicate that a ${\dot{\text{V}}\text{O}}_{{2{\text{pl}}}}$ and ${\dot{\text{V}}\text{O}}_{2\max }$ were reached in every single participant as demonstrated by the following example. Midgley et al. [77] did not find a significant difference in mean ${\dot{\text{V}}\text{O}}_{{2{\text{peak}}}}$ values between the incremental and verification tests (Table 1). Analysing the 32 incremental tests, only 16 ${\dot{\text{V}}\text{O}}_{{2{\text{pl}}}}$s were found. In contrast, ${\dot{\text{V}}\text{O}}_{2\max }$ of 26 out of the 32 incremental tests was verified by a not considerably higher ${\dot{\text{V}}\text{O}}_{2}$ value (< 2%) in the verification bout, suggesting a ${\dot{\text{V}}\text{O}}_{{2{\text{pl}}}}$ incidence between the incremental and the verification bout of 81%. However, they [77] did not consider that in seven out of the 26 positive verification bouts the ${\dot{\text{V}}\text{O}}_{2}$ was more than 3% lower than the corresponding ${\dot{\text{V}}\text{O}}_{{2{\text{peak}}}}$ value of the incremental test. Midgley et al. [77] attributed this lower verification bout ${\dot{\text{V}}\text{O}}_{2}$values to insufficient verification bout durations. Therefore, it is unclear whether ${\dot{\text{V}}\text{O}}_{2\max }$ was achieved in the corresponding incremental tests. When subtracting these seven tests only 19 out of the 32 incremental tests (59%) were really verified by the verification bouts, which is close to the number of identified ${\dot{\text{V}}\text{O}}_{{2{\text{pl}}}}$'s via incremental tests only (50%). The latter example combined with the fact that most studies in Table 1 reported even shorter TTE values of the verification bouts indicate rather limited evidence for an advantage of supramaximal verification tests over single incremental test procedures in the determination of a ${\dot{\text{V}}\text{O}}_{{2{\text{pl}}}}$ and ${\dot{\text{V}}\text{O}}_{2\max }$.

In contrast, it has been shown that submaximal verification bouts (e.g. 95% of maximum work rate) may be useful to identify submaximal ${\dot{\text{V}}\text{O}}_{{2{\text{peak}}}}$ values of incremental tests, especially in sedentary [98] and clinical [99] populations, but also in recreationally active participants [42]. However, it is important to note that a submaximal verification bout does not allow constructing a ${\dot{\text{V}}\text{O}}_{{2{\text{pl}}}}$, since this is defined as a more or less constant ${\dot{\text{V}}\text{O}}_{2}$ despite an increase in work rate [7, 11]. As a result, one cannot be certain that ${\dot{\text{V}}\text{O}}_{2\max }$ has been achieved when the ${\dot{\text{V}}\text{O}}_{{2{\text{peak}}}}$ of a submaximal verification bout is similar to ${\dot{\text{V}}\text{O}}_{{2{\text{peak}}}}$ of an incremental test. However, if the ${\dot{\text{V}}\text{O}}_{{2{\text{peak}}}}$ of a submaximal verification bout is higher than the ${\dot{\text{V}}\text{O}}_{{2{\text{peak}}}}$ of an incremental test one can be certain that ${\dot{\text{V}}\text{O}}_{2\max }$ has not been reached during the incremental phase. Furthermore, the higher ${\dot{\text{V}}\text{O}}_{{2{\text{peak}}}}$ of the submaximal verification bout is closer to ${\dot{\text{V}}\text{O}}_{2\max }$. Therefore, submaximal verification bouts, which can be sustained for sufficient durations, seem to be useful for the diagnosis of ${\dot{\text{V}}\text{O}}_{{2{\text{peak}}}}$, but not for the diagnosis of a ${\dot{\text{V}}\text{O}}_{{2{\text{pl}}}}$ and ${\dot{\text{V}}\text{O}}_{2\max }$.

## Physiological Determinants

As described in the previous sections it seems to be very likely that the ${\dot{\text{V}}\text{O}}_{{2{\text{pl}}}}$ is not simply a calculation artefact, as suggested by Beltrami et al. [32] and others [20, 24, 31, 33, 47]. However, there is also no convincing evidence for the assumption [11, 14, 34–36] that the absence of a ${\dot{\text{V}}\text{O}}_{{2{\text{pl}}}}$ is mostly caused in inappropriate exercise modes and protocols or data analysing approaches. Thus, irrespective of the exercise modes and protocols approximately 20–60% of the participants show a plateau at ${\dot{\text{V}}\text{O}}_{2\max }$ when probably valid plateau definitions have been used [18, 19, 42, 49–52]. This raises the question of why some participants demonstrate a ${\dot{\text{V}}\text{O}}_{{2{\text{pl}}}}$ and others do not.

### Why Does a ${\dot{\text{V}}\text{O}}_{2}$-Plateau Occur?

Hill and Lupton [3] interpreted the flattening of the ${\dot{\text{V}}\text{O}}_{2}$–running speed relationship at high running speeds as an indication that “the heart, lungs, circulation, and the diffusion of oxygen to the active muscle-fibres have attained their maximum activity.” In a subsequent publication they identified the capacity of the cardiovascular and respiratory systems to transport O_2_ as the main limiting factor of ${\dot{\text{V}}\text{O}}_{2\max }$ [1, 21]. The assumption that the ${\dot{\text{V}}\text{O}}_{{2{\text{pl}}}}$ reflects the upper limit of the cardiovascular and respiratory system to transport O_2_ to the muscles is supported by more recent studies [60, 100–102]. Brink-Elfegoun et al. [102] compared ${\dot{\text{V}}\text{O}}_{2}$and cardiac output (CO) using the Fick-principle during a constant load bout which barely elicits ${\dot{\text{V}}\text{O}}_{2\max }$ and during a 10–15% higher constant work rate. Despite the difference in work rate and sufficient duration of both constant load bouts, they found no differences in ${\dot{\text{V}}\text{O}}_{2\max }$ and CO between the higher and lower work rate bout. This indicates that the flattening of the ${\dot{\text{V}}\text{O}}_{2}$–work rate relationship at ${\dot{\text{V}}\text{O}}_{2\max }$is caused by the inability of the heart to further increase the rate of O_2_ delivery to the muscles. The finding of Brink-Elfegoun et al. [102] is supported by an older study from Miyamoto et al. [100] that described a close correlation between the work rates at which CO and ${\dot{\text{V}}\text{O}}_{2}$ start to level off.

Harms et al. [101] showed that in female participants with a considerable arterial O_2_ desaturation (≤ 92%) at ${\dot{\text{V}}\text{O}}_{2\max }$ an increase of the O_2_ partial pressure by breathing hyperoxic air (26% O_2_ and 74% N_2_) leads to an increase of ${\dot{\text{V}}\text{O}}_{2\max }$ and removal of a ${\dot{\text{V}}\text{O}}_{{2{\text{pl}}}}$ which was present at normoxic conditions. In contrast, participants without or only a slightly arterial O_2_ desaturation (≥ 93%) at ${\dot{\text{V}}\text{O}}_{2\max }$ showed no increase in ${\dot{\text{V}}\text{O}}_{2\max }$ when hyperoxic air was applied. Furthermore, they showed a ${\dot{\text{V}}\text{O}}_{{2{\text{pl}}}}$ in both conditions [101].

Based on these findings it seems to be very likely that the ${\dot{\text{V}}\text{O}}_{{2{\text{pl}}}}$ is caused by the achievement of the highest rate at which the body can transport (and utilise) ${\dot{\text{V}}\text{O}}_{2}$. This means that a real ${\dot{\text{V}}\text{O}}_{{2{\text{pl}}}}$ is caused by the same mechanisms that limit ${\dot{\text{V}}\text{O}}_{2\max }$, which are still controversially discussed [10, 22, 24, 33, 103–107]. However, for the occurrence of a ${\dot{\text{V}}\text{O}}_{{2{\text{pl}}}}$ it does not matter if ${\dot{\text{V}}\text{O}}_{2\max }$ is limited by the diffusion of O_2_ in the lung, the transport of O_2_ by the cardiovascular system or the utilisation of O_2_ in the muscles [103]. The crucial factor for the ${\dot{\text{V}}\text{O}}_{{2{\text{pl}}}}$ occurrence is that the mode-specific maximal rate of oxidative ATP generation (i.e. ${\dot{\text{V}}\text{O}}_{2\max }$) is reached before exercise is terminated due to fatigue or low levels of pain tolerance [49, 50, 103, 108, 109]. As described in Sect. 2.1, ${\dot{\text{V}}\text{O}}_{2\max }$ must be sustained for rather large increases in work rate (~ 40–50 W) to enable ${\dot{\text{V}}\text{O}}_{{2{\text{pl}}}}$ diagnoses with sufficient certainty [42]. This raises the question of what kind of ability enables some participants to tolerate further increases in work rate or speed after ${\dot{\text{V}}\text{O}}_{2\max }$ has been achieved.

### Motivation and Pain Tolerance

Exercise in the heavy- and severe-intensity domain goes along with the perception of discomfort and pain. Therefore, the achievement of a ${\dot{\text{V}}\text{O}}_{{{\text{2pl}}}}$ has been ascribed to a high-level motivation and pain tolerance [2, 23, 35–37, 103, 109]. In fact, some studies (two of them with a probably valid ${\dot{\text{V}}\text{O}}_{{{\text{2pl}}}}$definition [50, 51]) reported that the occurrence of a ${\dot{\text{V}}\text{O}}_{{{\text{2pl}}}}$ is accompanied by higher secondary exhaustion criteria, such as HR_max_, RER_max_ and BLC_max_ [50, 51, 109]. However, most other studies (three of them with a probably valid ${\dot{\text{V}}\text{O}}_{{{\text{2pl}}}}$ definition [18, 19, 49]) reported no significant differences in secondary exhaustion criteria between plateauing and non-plateauing participants [15, 18, 19, 49, 73, 110–112]. This indicates that a high level of motivation and pain tolerance is only a necessary but not a sufficient requirement for the achievement of a ${\dot{\text{V}}\text{O}}_{{{\text{2pl}}}}$. The latter conclusion is supported by a study of Doherty et al. [12] that found rather low ${\dot{\text{V}}\text{O}}_{{{\text{2pl}}}}$ incidences in elite runners, despite this cohort being accustomed to the discomfort and pain which goes along with high intensive exercise.

### Anthropometric, Age and Gender Determinants

It is widely believed that children are less likely to demonstrate a ${\dot{\text{V}}\text{O}}_{{{\text{2pl}}}}$ compared to adults [2, 69, 73, 93, 112, 113]. This assumption is based on studies [58, 69, 73, 110], which tried to measure ${\dot{\text{V}}\text{O}}_{{{\text{2max}}}}$ in children and found considerable lower ${\dot{\text{V}}\text{O}}_{{{\text{2pl}}}}$ incidences than the classical study of Taylor et al. [7]. As described in Sect. 2.3.2, it is very likely, that the extremely high ${\dot{\text{V}}\text{O}}_{{{\text{2pl}}}}$ incidence in the study of Taylor et al. [7] is caused by the specific approach of this study, which results in a high rate of false-positive ${\dot{\text{V}}\text{O}}_{{{\text{2pl}}}}$ diagnoses [5, 52]. Compared to studies with a probably valid methodological approach [18, 19, 42, 49–52] the reported ${\dot{\text{V}}\text{O}}_{{{\text{2pl}}}}$ incidences in children are mostly in the range of adult participants (i.e. 20–60%). However, to the best of our knowledge, there is not a single study that directly compared the ${\dot{\text{V}}\text{O}}_{{{\text{2pl}}}}$ incidences of children and adults. Studies comparing young participants with and without a ${\dot{\text{V}}\text{O}}_{{{\text{2pl}}}}$ found no differences in age [16, 114, 115]. According to Edvardsen et al. [17] there was also no effect of age on the ${\dot{\text{V}}\text{O}}_{{{\text{2pl}}}}$ incidence in a large cohort of more than 850 adult participants. Importantly, three of these studies used either an uncommon ${\dot{\text{V}}\text{O}}_{{{\text{2pl}}}}$ definition (no increase in ${\dot{\text{V}}\text{O}}_{{2}}$ despite an increase in V̇E) [17] or fixed cut-offs without reporting the corresponding sampling intervals [16, 114]. As a result, it is unclear whether the findings of these studies are valid.

Beside of age, several studies observed the effect of height, body mass, body mass index, and sex on the probability of the ${\dot{\text{V}}\text{O}}_{{{\text{2pl}}}}$ occurrence. Only one study, which used a probably inappropriate ${\dot{\text{V}}\text{O}}_{{{\text{2pl}}}}$ definition, described a significantly higher ${\dot{\text{V}}\text{O}}_{{{\text{2pl}}}}$ incidence in female compared to male adults [116]. All other studies found no systematic effects of height, body mass, body mass index, and sex on the ${\dot{\text{V}}\text{O}}_{{{\text{2pl}}}}$ incidence [15–17, 69, 110, 112, 114, 115, 117]. Despite the validity of the ${\dot{\text{V}}\text{O}}_{{{\text{2pl}}}}$ definitions of some of these studies is unclear [16, 110, 112, 114, 116, 117], the large amount of nearly consistent evidence indicates that the ${\dot{\text{V}}\text{O}}_{{{\text{2pl}}}}$ occurrence is likely not affected by sex and anthropometric measurements. In contrast, the present evidence is insufficient to assess whether the ${\dot{\text{V}}\text{O}}_{{{\text{2pl}}}}$ incidence differs between children and adults or depends on age.

### Aerobic Fitness and Endurance Training

It is partly believed that (endurance) trained athletes are more likely to show a plateau at ${\dot{\text{V}}\text{O}}_{{{\text{2max}}}}$ [2, 35, 36, 58, 103, 118]. This is based on the assumption that athletes are accustomed to sustained high-intensity exercise [103]. However, there is only one single study that compared the ${\dot{\text{V}}\text{O}}_{{{\text{2pl}}}}$ incidences between endurance-trained and untrained participants [51]. This study reported a significantly higher ${\dot{\text{V}}\text{O}}_{{{\text{2pl}}}}$ incidence in world-class cyclists (47%) compared to healthy sedentary participants (24%). In contrast, another study on elite runners found rather low ${\dot{\text{V}}\text{O}}_{{{\text{2pl}}}}$ incidences in incremental exercise tests that were performed on a treadmill [12]. Furthermore, markers of the V̇O_2_pl occurrence are not correlated with ${\dot{\text{V}}\text{O}}_{{{\text{2max}}}} /{\dot{\text{V}}\text{O}}_{{{\text{2peak}}}}$ in most studies observing the ${\dot{\text{V}}\text{O}}_{{{\text{2pl}}}}$ incidence in heterogeneous cohorts [13, 15, 16, 19, 43, 110]. This is supported by studies that found no differences in ${\dot{\text{V}}\text{O}}_{{{\text{2max}}}} /{\dot{\text{V}}\text{O}}_{{{\text{2peak}}}}$ between participants with and without a V̇O_2_pl [49, 50]. Furthermore, Gordon et al. [45] found a significantly lower ${\dot{\text{V}}\text{O}}_{{{\text{2max}}}}$ but equal V̇O_2_pl incidences after a blood donation compared to the pre-blood donation test. Despite some of these studies used ${\dot{\text{V}}\text{O}}_{{{\text{2pl}}}}$ definitions with unclear or questionable validity [12, 15, 16, 45, 110] this indicates that the ${\dot{\text{V}}\text{O}}_{{{\text{2pl}}}}$ occurrence is likely independent of aerobic fitness. Further studies are needed to verify whether endurance-trained participants that are accustomed to high-intensity exercise have higher ${\dot{\text{V}}\text{O}}_{{{\text{2pl}}}}$ incidences than untrained participants.

### Anaerobic Power and Capacity

Since energy demand above ${\dot{\text{V}}\text{O}}_{{{\text{2max}}}}$ must be matched by anaerobic adenosine triphosphate (ATP) generation, the occurrence of a ${\dot{\text{V}}\text{O}}_{{{\text{2pl}}}}$ has been ascribed to differences in anaerobic power and capacity [17, 111, 112]. Thus, several studies observed the effect of anaerobic measurements on the ${\dot{\text{V}}\text{O}}_{{{\text{2pl}}}}$ occurrence [43, 108, 115, 119–122]. Most of them checked whether the occurrence of a ${\dot{\text{V}}\text{O}}_{{{\text{2pl}}}}$ depends on measurements of anaerobic power or force, like jumping height, running sprint performance, isokinetic knee extension/flexion or peak power of a 30 s Wingate-test [43, 115, 119, 120, 122]. However, it seems to be very unlikely that the maximum work rate of an incremental exercise test is limited by anaerobic power. Thus, peak-power of a 30 s Wingate-test or a 6 s sprint-test is 2–4 times higher than maximum work rate of an incremental test, which is used to measure ${\dot{\text{V}}\text{O}}_{{{\text{2max}}}}$ [123, 124]. Consequently, it is not surprising that none of these studies could find a systematic difference between plateauing and non-plateauing participants [43, 115, 119, 120, 122]. The ability to sustain ${\dot{\text{V}}\text{O}}_{{{\text{2max}}}}$ for long durations is much more likely affected by anaerobic capacity than anaerobic power, as shown for constant load exercise [125–127].

**Fig. 5:**
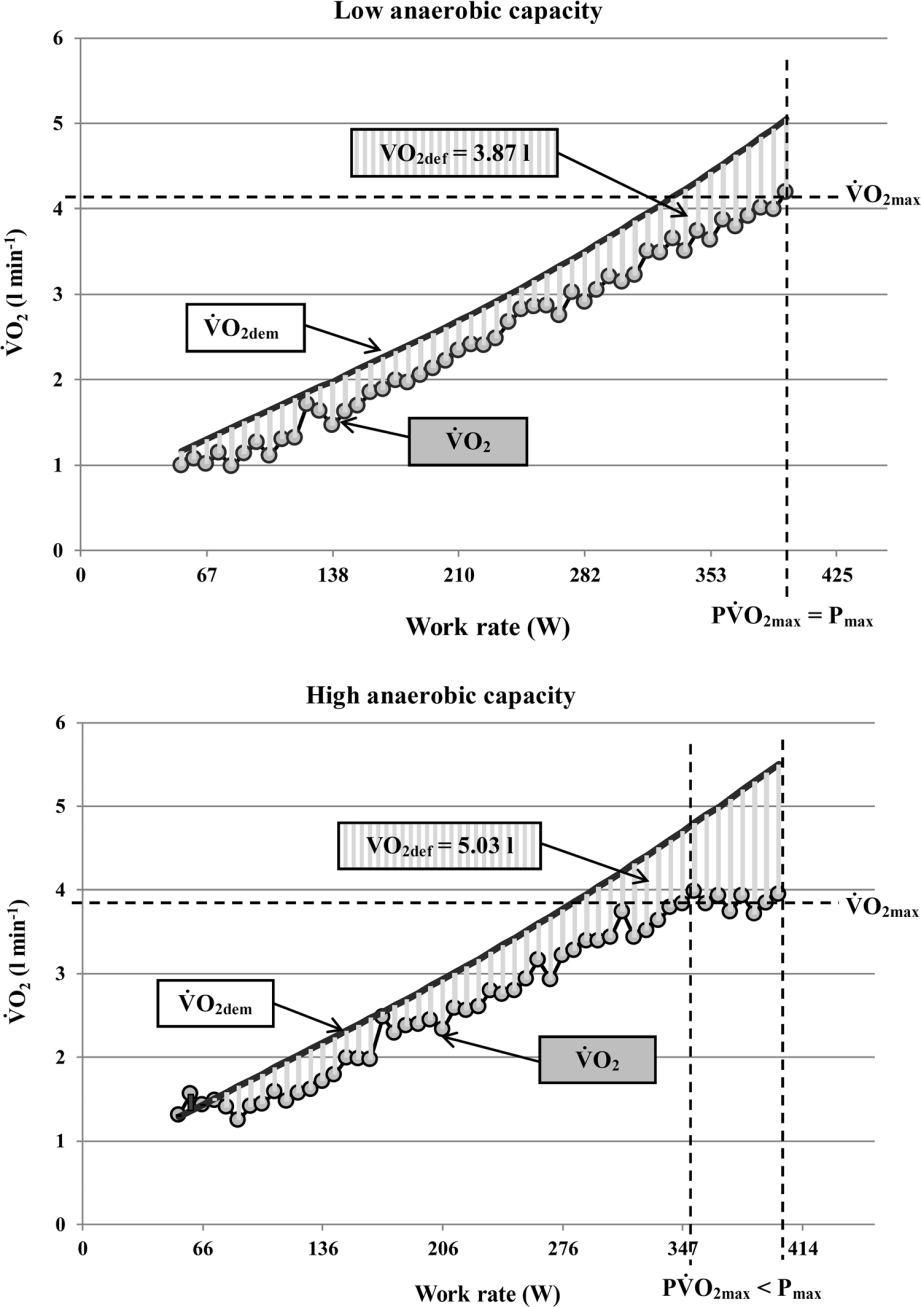
The effect of low vs. high anaerobic capacity on the oxygen uptake plateau (${\dot{\text{V}}\text{O}}_{{{\text{2pl}}}}$) occurrence in an incremental ramp test. Note that the difference between oxygen uptake demand (${\dot{\text{V}}\text{O}}_{{{\text{2dem}}}}$) and actual ${\dot{\text{V}}\text{O}}_{{{\text{2}}}}$ results in an accumulation of ${\dot{\text{V}}\text{O}}_{{{\text{2}}}}$ deficit (${\dot{\text{V}}\text{O}}_{{{\text{2def}}}}$). Higher anaerobic capacity in terms of a larger tolerable ${\dot{\text{V}}\text{O}}_{{{\text{2def}}}}$ enables to sustain a further increase in work rate after ${\dot{\text{V}}\text{O}}_{{{\text{2max}}}}$ has been achieved. As a result, the maximum work rate (*P*_max_) is higher than the work rate at the first achievement of ${\dot{\text{V}}\text{O}}_{{{\text{2max}}}}$ (P${\dot{\text{V}}\text{O}}_{{{\text{2max}}}}$), which leads to the occurrence of a ${\dot{\text{V}}\text{O}}_{{{\text{2pl}}}}$. Measured ${\dot{\text{V}}\text{O}}_{{2}}$ data are averaged over 10-s intervals for clarity

Based on these findings Gordon et al. [108] firstly checked whether the ${\dot{\text{V}}\text{O}}_{{{\text{2pl}}}}$ occurrence in incremental ramp tests depends on anaerobic capacity. They found a significant negative relationship between the maximal accumulated oxygen deficit and the increase in ${\dot{\text{V}}\text{O}}_{{2}}$ during the final minute of the ramp test, which indicates that participants with a higher anaerobic capacity are more likely to show a plateau at ${\dot{\text{V}}\text{O}}_{{{\text{2max}}}}$. As shown in Fig. 5, this is probably caused by the fact that during incremental exercise a continuous accumulation of ${\dot{\text{V}}\text{O}}_{{2}}$-deficit occurs due to a lagging behaviour of ${\dot{\text{V}}\text{O}}_{{2}}$ [49, 128, 129]. The ${\dot{\text{V}}\text{O}}_{{2}}$-deficit must be matched by anaerobic energy contribution. This leads to the accumulation of muscle metabolites and, therefore, to exercise termination when a maximum tolerable concentration of fatigue-inducing metabolites has been achieved [49, 128–133]. At a given ${\dot{\text{V}}\text{O}}_{{2}}$-deficit accumulation and ${\dot{\text{V}}\text{O}}_{{{\text{2max}}}}$, a higher anaerobic capacity should enable a higher maximum work rate in an incremental exercise test. Participants with a higher anaerobic capacity are therefore able to tolerate a further increase in work rate or speed after ${\dot{\text{V}}\text{O}}_{{{\text{2max}}}}$ has been achieved, which may result in the occurrence of a ${\dot{\text{V}}\text{O}}_{{{\text{2pl}}}}$, as shown in Fig. 5. This is supported by a recent study of Keiller and Gordon [134], which showed that the occurrence of the ${\dot{\text{V}}\text{O}}_{{{\text{2pl}}}}$ is associated with anaerobic alleles.

However, both studies [108, 134] used rather short sampling intervals (60 s or 60 breaths) combined with fixed cut-offs, such that the findings may be affected by elevated risks of false-positive and/or false-negative ${\dot{\text{V}}\text{O}}_{{{\text{2pl}}}}$ diagnoses. In addition, a more recent study by Silva et al. [121] did not find a correlation between the maximal accumulated oxygen deficit and measurements of the ${\dot{\text{V}}\text{O}}_{{{\text{2pl}}}}$ occurrence. This indicates that the ${\dot{\text{V}}\text{O}}_{{{\text{2pl}}}}$ occurrence is not solely affected by anaerobic capacity.

### Oxygen Uptake Kinetics

The ability to sustain high-intensity exercise for a long duration is additionally determined by ${\dot{\text{V}}\text{O}}_{{2}}$-kinetics [135, 136]. ${\dot{\text{V}}\text{O}}_{{2}}$-kinetics is defined as the rate at which aerobic ATP generation adjusts to a change of exercise intensity [82]. The faster the ${\dot{\text{V}}\text{O}}_{{2}}$-kinetics the lower the ${\dot{\text{V}}\text{O}}_{{2}}$-deficit and the related accumulation of anaerobic metabolites at the beginning of exercise [136–139]. Consequently, faster ${\dot{\text{V}}\text{O}}_{{2}}$-kinetics lowers the ${\dot{\text{V}}\text{O}}_{{2}}$-deficit and spares anaerobic capacity, which leads to higher exercise tolerance and time to exhaustion during constant or intermittent exercise [140–143]. Furthermore, participants with faster ${\dot{\text{V}}\text{O}}_{{2}}$-kinetics achieve their ${\dot{\text{V}}\text{O}}_{{{\text{2max}}}}$ earlier compared to counterparts with a slower ${\dot{\text{V}}\text{O}}_{{2}}$-kinetics [49, 81].

**Fig. 6:**
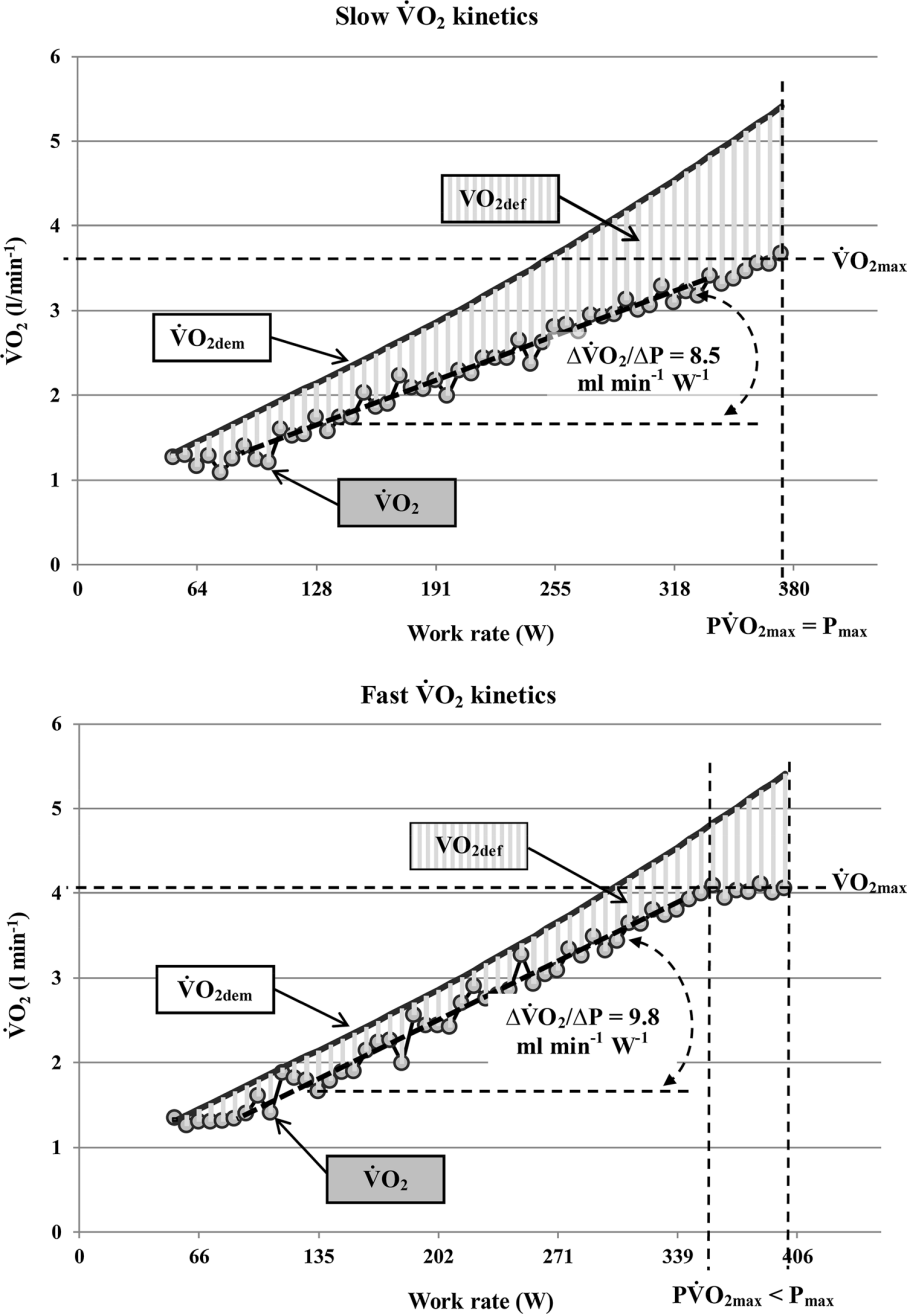
The effect of slow vs. fast oxygen uptake (${\dot{\text{V}}\text{O}}_{{{\text{2}}}}$) ramp test kinetics on the ${\dot{\text{V}}\text{O}}_{{{\text{2pl}}}}$ occurrence. Note that a faster ${\dot{\text{V}}\text{O}}_{{{\text{2}}}}$-kinetics which is reflected by a steeper increase in ${\dot{\text{V}}\text{O}}_{{{\text{2}}}}$ per increase in work rate (∆${\dot{\text{V}}\text{O}}_{{{\text{2}}}}$/∆P) results in a lower difference between ${\dot{\text{V}}\text{O}}_{{{\text{2}}}}$ demand (${\dot{\text{V}}\text{O}}_{{{\text{2dem}}}}$) and actual ${\dot{\text{V}}\text{O}}_{{{\text{2}}}}$ and, therefore, in lower ${\dot{\text{V}}\text{O}}_{{{\text{2}}}}$ deficit (V̇O_2def_) accumulation up to the work rate of maximum oxygen uptake (${\dot{\text{V}}\text{O}}_{{{\text{2max}}}}$) achievement (P${\dot{\text{V}}\text{O}}_{{{\text{2}}}}$). This enables the participant to sustain ${\dot{\text{V}}\text{O}}_{{{\text{2max}}}}$ despite a further increase in work rate and leads to the occurrence of a ${\dot{\text{V}}\text{O}}_{{{\text{2pl}}}}$ (for further details see Niemeyer et al. 2019). Measured ${\dot{\text{V}}\text{O}}_{{{\text{2}}}}$ data are averaged over 10-s intervals for clarity

The effect of ${\dot{\text{V}}\text{O}}_{{2}}$-kinetics on the ${\dot{\text{V}}\text{O}}_{{{\text{2pl}}}}$ occurrence has been described recently [49]. This study demonstrated that participants with a ${\dot{\text{V}}\text{O}}_{{{\text{2pl}}}}$ have faster ramp- and square-wave V̇O_2_-kinetics. As schematically shown in Fig. 6, the faster ${\dot{\text{V}}\text{O}}_{{2}}$-kinetics leads to a lower ${\dot{\text{V}}\text{O}}_{{2}}$-deficit accumulation in the submaximal intensity domain and results in an earlier achievement of ${\dot{\text{V}}\text{O}}_{{{\text{2max}}}}$. As a result, participants with a faster ${\dot{\text{V}}\text{O}}_{{2}}$-kinetics are able to sustain their ${\dot{\text{V}}\text{O}}_{{{\text{2max}}}}$ for longer durations, which increases the chance of a ${\dot{\text{V}}\text{O}}_{{{\text{2pl}}}}$ occurrence at the end of a ramp test [49].

Since ${\dot{\text{V}}\text{O}}_{{2}}$-kinetics is speeded by a bout of priming exercise in the heavy/severe-intensity domain [85, 86], this finding seems to be supported by a study showing an increase in the ${\dot{\text{V}}\text{O}}_{{{\text{2pl}}}}$ incidence from 50 to 100% in ramp tests performed in a primed state compared to not primed ramp tests [40]. However, the authors used an inappropriate cut-off to check for the occurrence of a ${\dot{\text{V}}\text{O}}_{{{\text{2pl}}}}$, which likely led to a high rate of false-positive ${\dot{\text{V}}\text{O}}_{{{\text{2pl}}}}$ diagnoses [42, 144]. Furthermore, a subsequent study did not find higher ${\dot{\text{V}}\text{O}}_{{{\text{2pl}}}}$ incidences in primed compared to not primed ramp tests [144]. Since the latter study found no speeding of ramp test ${\dot{\text{V}}\text{O}}_{{2}}$-kinetics as well, this finding did neither support nor disprove a potential effect of ${\dot{\text{V}}\text{O}}_{{2}}$-kinetics on the incidence of a ${\dot{\text{V}}\text{O}}_{{{\text{2pl}}}}$ [144].

In conclusion, evidence from a comparison of participants with and without a ${\dot{\text{V}}\text{O}}_{{{\text{2pl}}}}$ suggests that ${\dot{\text{V}}\text{O}}_{{2}}$-kinetics is a determinant of the ${\dot{\text{V}}\text{O}}_{{2}}$-plateau occurrence. However, the cause–effect relationship needs to be proven by experimental research designs, which for example leads to speeding of ${\dot{\text{V}}\text{O}}_{{2}}$ ramp test kinetics, like priming exercise [145] or dietary nitrate supplementation [146].

### Accumulation of Anaerobic Metabolites

Another factor that has been considered as a major determinant of the ability to sustain high intensity exercise is the relation of the work rate at which lactate begins to accumulate and the work rate that elicits ${\dot{\text{V}}\text{O}}_{{{\text{2max}}}}$ [147–149]. This assumption is supported by significant negative relationships between lactate/ventilation thresholds expressed in %${\dot{\text{V}}\text{O}}_{{{\text{2max}}}}$ and time to exhaustion at the minimum running velocity that elicits ${\dot{\text{V}}\text{O}}_{{{\text{2max}}}}$ [126, 149, 150].

Lacour et al. [50] analysed blood lactate and ${\dot{\text{V}}\text{O}}_{{2}}$ values of stepwise incremental tests of 94 elite oarsmen, retrospectively. They found significantly lower blood lactate values in the submaximal intensity domain in the ${\dot{\text{V}}\text{O}}_{{{\text{2pl}}}}$ group. As a result, the 4 mmol L^−1^ lactate threshold occurred at a significantly higher percentage of ${\dot{\text{V}}\text{O}}_{{{\text{2max}}}}$ in the plateau compared to the non-plateau group. Furthermore, the %${\dot{\text{V}}\text{O}}_{{{\text{2max}}}}$ at the lactate threshold was closely negative correlated with the blood lactate concentration at the work rate step that firstly elicits ${\dot{\text{V}}\text{O}}_{{{\text{2max}}}}$. This seems to indicate that the oarsmen with a ${\dot{\text{V}}\text{O}}_{{{\text{2pl}}}}$ are able to spare their anaerobic capacity to a greater extent because of a higher anaerobic threshold expressed in %${\dot{\text{V}}\text{O}}_{{{\text{2max}}}}$ compared to the non-plateauing oarsmen. The sparing of anaerobic capacity seems to enable the ${\dot{\text{V}}\text{O}}_{{{\text{2pl}}}}$ group to tolerate a larger increase in work rate after ${\dot{\text{V}}\text{O}}_{{{\text{2max}}}}$ has been achieved, which leads to the occurrence of a ${\dot{\text{V}}\text{O}}_{{{\text{2pl}}}}$ [50].

### Central Governor

Based on low ${\dot{\text{V}}\text{O}}_{{{\text{2pl}}}}$ incidences Noakes [20, 24, 33] questioned the limitation of ${\dot{\text{V}}\text{O}}_{{{\text{2max}}}}$ and exercise performance by the provision of energy through aerobic and anaerobic ATP generation. Instead, he proposed a model in which the brain (central governor) regulates the recruitment of motor units to prevent damage of organs due to O_2_ deficiency or loss of homeostasis [151]. The extensive but seemingly inconclusive discussion about the validity of the central governor model goes beyond the scope of the present review. The interested reader is referred to a substantial, however, clearly not complete body of related publications [20–22, 24, 33, 35, 152, 153].

Nevertheless, Noakes [20, 24, 33] stated repeatedly that in the absence of a ${\dot{\text{V}}\text{O}}_{{{\text{2pl}}}}$termination of incremental exercise cannot be explained by the accumulation of anaerobic metabolites (muscle anaerobiosis). He strongly expressed the critique that since more than 20 years traditional concepts of exercise physiology failed to answer the following crucial question: “What causes the termination of exercise when the ‘‘true’’ ${\dot{\text{V}}\text{O}}_{{{\text{2max}}}}$ is achieved without the ‘‘plateau phenomenon’’?” [24]. Based on this supposedly unanswered question he concluded that … “the absence of the ‘‘plateau phenomenon’’ in a majority of ${\dot{\text{V}}\text{O}}_{{{\text{2max}}}}$ tests can logically be interpreted in only one way: that factors other than a limiting cardiac output and the development of skeletal muscle anaerobiosis must cause the termination of exercise in the majority of ${\dot{\text{V}}\text{O}}_{{{\text{2max}}}}$ tests” [33]. This conclusion is based on the assumption that “the absence of a plateau indicates adequate muscle oxygenation during maximal exercise” [33], which would exclude any kind of oxygen deficit accumulation and also net anaerobic energy contribution, as long as no ${\dot{\text{V}}\text{O}}_{{{\text{2pl}}}}$ occurs.

However, the assumption that the absence of a ${\dot{\text{V}}\text{O}}_{{{\text{2pl}}}}$ indicates adequate muscle oxygenation per se and no need for muscular anaerobic energy provision reflect a misinterpretation of energy metabolism. As described in the previous sections there is a continuous accumulation of O_2_-deficit from the beginning of an incremental exercise test due to a lagging behaviour of ${\dot{\text{V}}\text{O}}_{{2}}$ [49, 128, 129, 132]. Furthermore, at work rates above maximal lactate steady-state or critical power metabolic energy is partly provided by anaerobic ATP-generation, which leads to the accumulation of anaerobic metabolites and muscular fatigue [130, 131, 146, 154–156]. Therefore, the termination of incremental exercise despite the absence of a ${\dot{\text{V}}\text{O}}_{{{\text{2pl}}}}$ can be well explained by ${\dot{\text{V}}\text{O}}_{{2}}$-deficit accumulation, anaerobic energy contribution and the resulting accumulation of anaerobic metabolites [49, 50, 108, 128, 129, 148]. There is no need for a central governor to explain the presence or absence of a ${\dot{\text{V}}\text{O}}_{{{\text{2pl}}}}$.

## Consequences for the Diagnosis of ${\dot{\text{V}}\text{O}}_{{{\text{2max}}}}$

As described in the introduction section one can be certain that ${\dot{\text{V}}\text{O}}_{{{\text{2max}}}}$ has been reached only if ${\dot{\text{V}}\text{O}}_{{{\text{2}}}}$ remains more or less constant despite an increase in work rate. Thus, the diagnosis of ${\dot{\text{V}}\text{O}}_{{{\text{2max}}}}$ requires—per definition—a ${\dot{\text{V}}\text{O}}_{{{\text{2pl}}}}$. However, inconsistencies in methodology and data processing in previous studies led to a lot of confusion about the correct diagnosis of the ${\dot{\text{V}}\text{O}}_{{{\text{2pl}}}}$. When appropriate ${\dot{\text{V}}\text{O}}_{{{\text{2pl}}}}$ definitions and methods have been used the incidence of the ${\dot{\text{V}}\text{O}}_{{{\text{2pl}}}}$ during incremental exercise tests is usually less than 60% (see Sect. 2.1). Furthermore, there is no convincing evidence for the assumptions that a ${\dot{\text{V}}\text{O}}_{{{\text{2pl}}}}$ occurs in most participants when an incremental test is supplemented by a supramaximal verification bout or when a classical discontinuous exercise test is performed (see Sects. 2.3.2 and 2.3.3). This means that based on the present evidence ${\dot{\text{V}}\text{O}}_{{{\text{2max}}}}$ cannot be diagnosed in a considerable fraction of participants. Since this applies to often more than 50% of the participants it seems to be unreasonable to exclude all participants without a ${\dot{\text{V}}\text{O}}_{{{\text{2pl}}}}$. As a consequence, we have to accept the diagnosis of ${\dot{\text{V}}\text{O}}_{{{\text{peak}}}}$ at least until there is more substantial evidence that most participants achieve a ${\dot{\text{V}}\text{O}}_{{{\text{2pl}}}}$ by applying a specific methodological approach.

Nevertheless, even if ${\dot{\text{V}}\text{O}}_{{{\text{2max}}}}$ cannot be diagnosed, it is important that ${\dot{\text{V}}\text{O}}_{{{\text{peak}}}}$ equals or is at least close to ${\dot{\text{V}}\text{O}}_{{{\text{2max}}}}$. Otherwise, the efficiency of training interventions cannot be evaluated with sufficient certainty [11, 19]. Furthermore, a ${\dot{\text{V}}\text{O}}_{{{\text{peak}}}}$ which is considerably less than ${\dot{\text{V}}\text{O}}_{{{\text{2max}}}}$ may lead to fatal misdiagnoses since ${\dot{\text{V}}\text{O}}_{{{\text{peak}}}}$ is an important marker in clinical sports medicine and cardiology [157, 158]. The most common strategy to ensure that ${\dot{\text{V}}\text{O}}_{{{\text{peak}}}}$ is close to ${\dot{\text{V}}\text{O}}_{{{\text{2max}}}}$ is to apply secondary exhaustion criteria, like maximum blood lactate concentration, maximum heart rate, maximum respiratory exchange ratio or rating of or maximum rating of perceived exertion [2, 23, 25]. They can be used to reduce the magnitude of a potential underestimation of ${\dot{\text{V}}\text{O}}_{{{\text{2max}}}}$, as recently described by Knaier et al. [18] and Wagner et al. [19]. However, the values of these criteria vary considerably between participants [37, 75] and are affected by the exercise protocol used [52, 62, 99]. Therefore, even if rather high and age-adjusted secondary exhaustion criteria are used an underestimation of ${\dot{\text{V}}\text{O}}_{{{\text{2max}}}}$ cannot be excluded [98]. Another strategy is to perform submaximal verification bouts (e.g. 95% of maximum work rate) after a common incremental test. As recently demonstrated this approach may be useful to identify submaximal ${\dot{\text{V}}\text{O}}_{{{\text{peak}}}}$ values of incremental tests, especially in sedentary and clinical populations [98, 99], but also in recreationally active participants [42]. However, as described in Sect. 2.3.3 submaximal verification bouts do not allow for the diagnosis of a ${\dot{\text{V}}\text{O}}_{{{\text{2pl}}}}$ and corresponding ${\dot{\text{V}}\text{O}}_{{{\text{2max}}}}$.

Based on this and the previous sections we recommend the following approach: For most studies in which ${\dot{\text{V}}\text{O}}_{{{\text{2max}}}} /{\dot{\text{V}}\text{O}}_{{{\text{2peak}}}}$ serves as a descriptive variable (like, age or sex) only, it seems to be sufficient to verify ${\dot{\text{V}}\text{O}}_{{{\text{2peak}}}}$ by the use of adequate and age-adjusted secondary exhaustion criteria [18, 19]. For all studies in which ${\dot{\text{V}}\text{O}}_{{{\text{2max}}}} /{\dot{\text{V}}\text{O}}_{{{\text{2peak}}}}$ is a main outcome, the ${\dot{\text{V}}\text{O}}_{{{\text{2}}}}$ profile should be checked for the occurrence of a ${\dot{\text{V}}\text{O}}_{{{\text{2pl}}}}$ by setting the cut-off at 50% of the increase in the submaximal intensity domain and using rather large sampling intervals (see Sect. 2.1). If no ${\dot{\text{V}}\text{O}}_{{{\text{2pl}}}}$ occurs a (submaximal) verification test should be performed, which allows for a sufficient duration of the verification bout (see Sect. 2.3.3) to ensure that ${\dot{\text{V}}\text{O}}_{{{\text{2peak}}}}$ is close as possible to ${\dot{\text{V}}\text{O}}_{{{\text{2max}}}}$. This seems to be especially recommendable in sedentary and clinical populations [98, 99] In contrast, for highly endurance-trained participants this approach seems to be an insufficient because they do not achieve ${\dot{\text{V}}\text{O}}_{{{\text{2max}}}}$ during short-lasting constant load tests [95–97].

## Conclusions

A substantial fraction of studies used inappropriate ${\dot{\text{V}}\text{O}}_{{{\text{2pl}}}}$ definitions and approaches such that the validity of their findings is limited. As a result, it is unclear whether the ${\dot{\text{V}}\text{O}}_{{{\text{2pl}}}}$incidence depends on exercise mode or incremental rate (see Sects. 2.2 and 2.3.1). Furthermore, there is no convincing evidence for the assumption that there is a higher ${\dot{\text{V}}\text{O}}_{{{\text{2pl}}}}$ incidence in classically discontinuous tests compared to continuous incremental tests (see Sect. 2.3.2). Since most studies that evaluated supramaximal verification phases, reported insufficient durations of the verification bouts, there is also rather limited evidence for an advantage of supramaximal verification tests approaches over single incremental test procedures in the determination of a ${\dot{\text{V}}\text{O}}_{{{\text{2pl}}}}$ and ${\dot{\text{V}}\text{O}}_{{{\text{2max}}}}$ (see Sect. 2.3.3). Preliminary evidence suggests that the occurrence of a ${\dot{\text{V}}\text{O}}_{{{\text{2pl}}}}$ is determined by physiological factors like anaerobic capacity, ${\dot{\text{V}}\text{O}}_{{2}}$-kinetics and accumulation of metabolites in the submaximal intensity domain.

Subsequent studies should take more attention to the use of valid ${\dot{\text{V}}\text{O}}_{{{\text{2pl}}}}$ definitions to avoid that their findings are biased by a high risk of false ${\dot{\text{V}}\text{O}}_{{{\text{2pl}}}}$ diagnoses. Therefore, cut-offs should be set at 50% of the corresponding increase in ${\dot{\text{V}}\text{O}}_{{2}}$ in the submaximal intensity domain and rather large sampling intervals should be used to enable an equal risk of false-positive and false-negative ${\dot{\text{V}}\text{O}}_{{{\text{2pl}}}}$ diagnoses as well as to account for the ventilation-induced variability of ${\dot{\text{V}}\text{O}}_{{2}}$ (see Sect. 2.1). If verification bouts are used to verify the achievement of ${\dot{\text{V}}\text{O}}_{{{\text{2max}}}} /{\dot{\text{V}}\text{O}}_{{{\text{2peak}}}}$, it should be ensured that they can be sustained for sufficient durations to enable ${\dot{\text{V}}\text{O}}_{{2}}$ to rise to the maximum value.
